# Supplementary material for: Governance for injury care systems in Ghana, South Africa and Rwanda: development and pilot testing of an assessment tool
Source: BMJ Open. 2023 Sep 4;13(9):e074088. doi: 10.1136/bmjopen-2023-074088 (PMC10481730; doi:10.1136/bmjopen-2023-074088)
Supplement: Supplementary data [file bmjopen-2023-074088supp001.pdf]

Appendix table 1. Copy of the governance survey

Principle	Questions	Responses (please circle one response for each question)	Maximum score
**Strategic vision**	Is there a specific mention of trauma in the national health plan or policy? Or are there specific national health policies around trauma	Don't know<br>No (0)<br>Yes (1)	**1**
	Is there a national trauma strategy?	Don't know<br>No (0)<br>Yes (1)	1
	What percentage of the country's health budget goes to trauma care?	Don't know<br><10% ((0)<br>10-20% (1)<br>>20% (2)	2
	Is public sector trauma care provided free of charge	Don't know<br>No-not at all (0)<br>Partly (1)<br>Yes-fully (2)	2
	Is there a national insurance scheme that covers trauma care	Don't know<br>No-not at all (0)<br>Partly (1)<br>Yes-fully (2)	2
	Is there a department within the National Ministry of Health dedicated for trauma?	Don't know<br>No department for trauma at all (0)<br>Department for trauma included in another department (1)<br>Stand-alone department for trauma (2)	2
	If the National health plan mentions trauma, or if there are national health policies for trauma care, are there specific objectives	Don't know<br>No (0)	1

	relating to trauma and timelines to achieve them?	Yes (1)	
	If you have answered yes to question 7, have any of those specific trauma objectives been implemented?	Don't know<br>No (0)<br>Yes (1)	1
Scores			12
**Participation and consensus orientation**	Are stakeholders (e.g: NGOs, private companies) involved in health policy formulation for trauma?	Don't know<br>No organisation is involved (0)<br>1-3 organisations are involved (1)<br>> 3 organisations are involved (2)	2
	What is the level of stakeholder engagement/community participation at the national and provincial level in trauma policy and related interventions?	Don't know<br>No involvement at all (0)<br>These groups are involved (1)	1
Scores			3
**Rule of law**	Are there guidelines for accreditation of trauma care providers (doctors, nurses, etc) and are these enforced?	Don't know<br>No guidelines present (0)<br>Guidelines present are not enforced (1)<br>Guidelines present and enforced (2)	2
	Are there laws to enforce a duty of care by hospitals to treat uninsured trauma patients?	Don't know<br>No laws (0)<br>Laws present are not enforced (1)<br>Laws present and enforced (2)	2
	Are there laws to protect against trauma? Example Seat belt laws?	Don't know<br>No laws (0)<br>Laws present are not enforced (1)	2

		Laws present and enforced (2)	
Scores			6
**Transparency**	Is information readily available on financial commitments or allocated budget to trauma care in the public sector at either national or provincial level?	Don't know<br>Don't know<br>No (0)<br>Yes (1)	1
	Are managers (District Directors of Health, Medical Superintendents of hospitals) evaluated on their health facility or facilities reaching specific targets for trauma care? And if so, are the results of these evaluations available and accessible?	Don't know<br>No trauma related criteria for assessment (0)<br>Trauma related criteria for assessment is used, but not available (1)<br>Trauma related criteria for assessment is used and available (2)	2
Scores			3
**Responsiveness of institutions**	Is there mandatory reporting of health facility trauma data and is this used to define the burden of injury at a national level?	Don't know<br>No (1)<br>Some data (1)<br>Adequate/complete data (2)	2
	Are these data used to inform national or provincial policy?	Don't know<br>No (0)<br>Yes (1)	1
	Are data on clinical post-injury outcomes captured routinely by health facilities?	Don't know<br>No (0)<br>Yes partially captured (1)<br>Yes adequately captured (2)	2

	Are data on post -injury care patient satisfaction routinely captured?	Don't know<br><br>No (0)<br><br>Yes partially captured (1)<br><br>Yes adequately captured (2)	2
	Are these trauma data used in planning services?	Don't know<br><br>No (0)<br><br>Yes (1)	1
	What is the level of responsiveness of the health system to non-medical needs of injured people (social needs, mental health needs etc) ?	Don't know<br><br>Health services cater to or assess only medical needs (1)<br><br>Health services consider other outcomes relevant to patients, for example their opinions on services provided (2)	2
Scores			10
**Equity**	Are there national level financial schemes to ensure the poor who are injured do not have to pay out of pocket direct medical costs of trauma care?	Don't know<br><br>No scheme (0)<br><br>Partial scheme (1)<br><br>All people are covered (2)	2
	Are there health policies in place to address inequality in access to care for trauma?	Don't know<br><br>No policies in place (0)<br><br>Policies in place for general health equity (1)<br><br>Policies in place specifically for trauma (2)	2
	Are there data which show whether access is equitable or not? And do those data show that access is equitable?	Don't know<br><br>No data available (0)<br><br>Data available (1)<br><br>Data available and show that access is equitable (2)	2

	Is the allocation of trauma care staff to districts and hospitals appropriate and based on needs? Do districts in urban centres get more staff than districts in remote areas?	Don't know<br><br>Less than 2:1 (1)<br><br>More than 2:1 (0)	1
	Is there a mechanism to equitably distribute the budget for health or trauma care?	Don't know<br><br>No (0)<br><br>Yes (1)	1
Scores			8
**Effectiveness and efficiency**	Is there a national trauma registry (information management for trauma care)? Is it used? In both private and public?	Don't know<br><br>No (0)<br><br>Yes (1)	1
	Are there national or provincial guidelines for in -service training of staff on trauma care?	Don't know<br><br>No (0)<br><br>Yes (1)	1
	Is there a pre-hospital referral system (e.g: ambulance service)?	Don't know<br><br>No (0)<br><br>Yes (1)	1
	If pre-hospital referral systems are available, what has been the experience of patients regarding pre hospital referrals?	Don't know<br><br>Poor (0)<br><br>Fair (1)<br><br>Good (2)	2
	Are local transport operators (e.g: taxis) involved in transporting people to hospital?	Don't know<br><br>No (0)<br><br>Yes (1)	1
Scores			6
**Accountability**	Are there mechanisms to report failing trauma services to policy makers or regulatory authorities	Don't know<br><br>No (0)<br><br>Yes (1)	1
	Are there any mechanisms of correcting under performance of trauma services?	Don't know<br><br>No mechanisms (0)	2

		Mechanisms of reporting but no onwards mechanisms to improve quality (1)<br><br>Mechanisms of reporting and mechanisms to improve quality (2)	
Scores			3
**Intelligence/Information**	Do staff providing trauma services understand what data needs to be captured and do they have the right data capturing tools to enable them do this?	Don't know<br><br>No understanding and no tools (0)<br><br>Understanding but no or limited tools (1)<br><br>Understanding and useful tools (2)	2
Scores			2
**Ethics**	Is there any policy available for regulating trauma related research?	Don't know<br><br>No (0)<br><br>Yes (1)	1
	Are there any standard operating procedures in place to ensure quality and ethical trauma care for injured people?	Don't know<br><br>No (0)<br><br>Yes (1)	1
	Are there mechanisms in place in institutes for enforcing high ethical standards in the treatment of trauma patients	Don't know<br><br>No (0)<br><br>Yes (1)	1
Scores			3
Overall maximum score			56

Appendix 2 Investigator score for each question and percentage score for each principle and overall for Rwanda, Ghana and South Africa respectively

Principle	Questions	Maximum score for question	Rwanda score	Ghana score	South Africa score
**Strategic vision**	Is there specific mention of trauma in the national health plan or policy? Or are there specific national health policies around trauma care?	1	1	1	1
	Is there a national trauma strategy?	1	1	0	0
	What percentage of the country's public health budget goes to trauma care?	2	0	0	2
	Is public sector trauma care provided free of charge?	2	1	1	1
	Is there a national insurance scheme that covers trauma care?	2	2	1	1
	Is there a department within the National Ministry of Health dedicated for trauma?	2	1	1	1
	If the National health plan mentions trauma, or if there are national health policies for trauma care, are there specific objectives relating to trauma and timelines to achieve them?	1	1	0	0
	If you have answered yes to question 7, have any of those specific trauma objectives been implemented?	1	1	0	0
**Score for Principle (% of total possible)**		**12**	**8 (67%)**	**4 (33%)**	**6 (50%)**
**Participation and consensus**	Are stakeholders (e.g: NGOs, private companies) involved in	2	2	2	2

	What is the level of stakeholder engagement/community participation at the national and	1	1	1	1
**Score for Principle (% of total possible)**		**3**	**3 (100%)**	**3 (100%)**	**3 (100%)**
**Rule of law**	Are there guidelines for accreditation of trauma care	2	2	2	1
	Are there laws to enforce a duty of care by hospitals to treat uninsured	2	0	2	2
	Are there laws to protect against trauma? Example Seat belt laws?	2	2	1	2
**Score for Principle (% of total possible)**		**6**	**4 (67%)**	**5 (83%)**	**5 (83%)**
**Transparency**	Is information readily available on financial commitments or allocated budget to trauma care in the public sector at either national or provincial level?	1	1	0	0
	Are managers (District Directors of Health, Medical Superintendents of hospitals) evaluated on their health facility or facilities reaching specific targets for trauma care? And if so, are the results of these evaluations available and accessible?	2	2	0	0
**Score for Principle (% of total possible)**		**3**	**3 (100%)**	**0 (0%)**	**0 (0%)**
**Responsiveness of institutions**	Is there mandatory reporting of health facility trauma data and is	2	2	1	1
	Are these data used to inform national or provincial policy?	1	1	1	1
	Are data on clinical post-injury outcomes captured routinely by	2	2	1	0
	Are data on post-injury care patient satisfaction routinely captured?	2	0	0	0
	Are these trauma data used in planning services?	1	1	1	1
	What is the level of responsiveness of the health system to non-medical needs of injured people (social needs, mental health needs etc)?	2	2	1	2

**Score for Principle (% of total possible)**		**10**	**8 (80%)**	**5 (50%)**	**5 (50%)**
**Equity**	Are there national level financial schemes to ensure the poor who are injured do not have to pay out	2	2	1	2
	Are there health policies in place to address inequality in access to care for trauma?	2	1	1	1
	Are there data which show whether access is equitable or not? And do those data show that access is equitable?	2	0	0	0
	Is the allocation of trauma care staff to districts and hospitals appropriate and based on needs? Do districts in urban centres get more staff than districts in remote areas?	1	0	0	0
	Is there a mechanism to equitably distribute the budget for health or trauma care?	1	0	0	0
**Score for Principle (% of total possible)**		**8**	**3 (38%)**	**2 (25%)**	**3 (38%)**
**Effectiveness and efficacy**	Is there a national trauma registry (information management for trauma care)? Is it used? In both private and public?	1	1	0	0
	Are there national or provincial guidelines for in-service training of staff on trauma care?	1	1	1	1
	Is there a pre-hospital referral system (e.g: ambulance service)?	1	1	1	1
	If pre-hospital referral systems are available, what has been the experience of patients regarding pre-hospital referrals?	2	1	1	2
	Are local transport operators (e.g: taxis) involved in transporting	1	1	1	0
**Score for Principle (% of total possible)**		**6**	**5 (83%)**	**4 (67%)**	**4 (67%)**

**Accountability**	Are there mechanisms to report failing trauma services to policy	1	0	1	1
	Are there any mechanisms of correcting under performance of	2	1	0	2
**Score for Principle (% of total possible)**		**3**	**1 (33.3%)**	**1 (33.3%)**	**3 (100%)**
**Intelligence/information**	Do staff providing trauma services understand what data needs to be captured and do they have the right	3	1	1	1
**Score for Principle (% of total possible)**		**3**	**1 (33%)**	**1 (33%)**	**1 (33%)**
**Ethics**	Is there any policy available for regulating trauma related research?	1	1	1	1
	Are there any standard operating procedures in place to ensure quality and ethical trauma care for injured people?	1	1	0	1
	Are there mechanisms in place in institutes for enforcing high ethical standards in the treatment of trauma patients?	1	1	1	1
**Score for Principle (% of total possible)**		**3**	**3 (100%)**	**2 (67%)**	**3 (100%)**
**Overall Total (% maximum overall score)**		**56**	**39 (70%)**	**27 (48%)**	**33 (59%)**

Appendix table 3 Summary results by principle for each country individually including average score, investigator score, maximum score possible for each question and achieved percentage score (average score and investigator score).

		Rwanda				Ghana				South Africa			
**Principle**	**Maximum scores**	**Respondent scores (n, average)**	**Investigator scores (n)**	**% achieved (Respondent scores)**	**% achieved (Investigator scores)**	**Respondent scores (n, average)**	**Investigator scores (n)**	**% achieved (Respondent scores)**	**% achieved (Investigator scores)**	**Respondent scores (n, average)**	**Investigator scores (n)**	**% achieved (Respondent scores)**	**% achieved (Investigator scores)**
Strategic vision	12	8.05	8	67.1%	66.7%	2.07	4	17.30%	33.30%	4.67	6	38.9%	50.0%
Participation and consensus orientation	3	3	3	100.0%	100.0%	1.4	3	46.70%	100%	3	3	100.0%	100.0%
Rule of law	6	4.75	4	79.2%	66.7%	3.16	5	63.20%	83.30%	4.83	5	80.5%	83.3%
Transparency	3	1.8	3	60.0%	100.0%	0.49	0	16.30%	0%	0	0	0.0%	0.0%
Responsiveness of institutions	10	7.65	8	76.5%	80.0%	3.54	5	35.40%	50%	5.25	5	52.5%	50.0%
Equity	8	2.95	3	36.9%	37.5%	1.4	2	17.50%	25%	3.23	3	40.4%	37.5%
Effectiveness and efficiency	6	5.35	5	89.2%	83.3%	3.28	4	54.70%	66.70%	4	4	66.7%	66.7%
Accountability	3	2.1	1	70.0%	33.3%	0.86	1	28.70%	33.30%	2.67	3	89.0%	100.0%
Intelligence/information	2	1.6	1	80.0%	50.0%	0.73	1	36.50%	50%	0.75	1	37.5%	50.0%
Ethics	3	2.6	3	86.7%	100.0%	1.57	2	52.30%	66.70%	2.67	3	89.0%	100.0%
**Overall score**	**56**	**39.85**	**39**	**71.2%**	**69.6%**	**18.5**	**27**	**33.00%**	**48.20%**	**31.07**	**33**	**55.5%**	**58.9%**
