# Supplementary material for: Governance for injury care systems in Ghana, South Africa and Rwanda: development and pilot testing of an assessment tool
Source: BMJ Open. 2023 Sep 4;13(9):e074088. doi: 10.1136/bmjopen-2023-074088 (PMC10481730; doi:10.1136/bmjopen-2023-074088)
Supplement: Author's manuscript [file bmjopen-2023-074088.draft_revisions.pdf]

BMJ Open is committed to open peer review. As part of this commitment we make the peer review history of every article we publish publicly available.

When an article is published we post the peer reviewers' comments and the authors' responses online. We also post the versions of the paper that were used during peer review. These are the versions that the peer review comments apply to.

The versions of the paper that follow are the versions that were submitted during the peer review process. They are not the versions of record or the final published versions. They should not be cited or distributed as the published version of this manuscript.

BMJ Open is an open access journal and the full, final, typeset and author-corrected version of record of the manuscript is available on our site with no access controls, subscription charges or pay-per-view fees (http://bmjopen.bmj.com).

If you have any questions on BMJ Open's open peer review process please email info.bmjopen@bmj.com

BMJ Open

Governance for injury care systems in Ghana, South Africa and Rwanda: development and pilot testing of an assessment tool

Journal:	*BMJ Open*
Manuscript ID	bmjopen-2023-074088
Article Type:	Original research
Date Submitted by the Author:	03-May-2023
Complete List of Authors:	The Equi-Trauma, Collaborative; University of Birmingham Institute of Applied Health Research<br>Odland, Maria Lisa; University of Birmingham, Institute of Applied Health Research; St Olavs Hospital Trondheim University Hospital, Department of Obstetrics and Gyneacology<br>Abdul-Latif , Abdul-Malik ; Ghana Health Service, Volta Regional Health Directorate<br>Ignatowicz, Agnieszka ; University of Birmingham Institute of Applied Health Research<br>Bekele, Abebe; University of Global Health Equity<br>Chu, Kathryn; Stellenbosch University Faculty of Medicine and Health Sciences, Centre for Global Surgery, Department of Global Health; University of Botswana, Department of Surgery<br>Howard, Anthony; University of Leeds Leeds Institute of Rheumatic and Musculoskeletal Medicine; University of Oxford, Nuffield Department of Orthopaedics, Rheumatology and Musculoskeletal Sciences, National Institute of Health Research (NIHR) Biomedical Centre<br>Stephen, Tabiri; Ghana Hub of NIHR Global Surgery; Tamale Teaching Hospital, Department of Surgery<br>BYIRINGIRO, JEAN CLAUDE; University of Rwanda College of Medicine and Health Sciences; University Teaching Hospital of Kigali, Department of Surgery<br>Davies, Justine ; University of Birmingham, Institute of Applied Health Research; Stellenbosch University Faculty of Medicine and Health Sciences, Centre for Global Surgery, Department of Global Health
Keywords:	Health policy < HEALTH SERVICES ADMINISTRATION & MANAGEMENT, Trauma management < ORTHOPAEDIC & TRAUMA SURGERY, ACCIDENT & EMERGENCY MEDICINE, Clinical governance < HEALTH SERVICES ADMINISTRATION & MANAGEMENT

SCHOLARONE™
Manuscripts

I, the Submitting Author has the right to grant and does grant on behalf of all authors of the Work (as defined in the below author licence), an exclusive licence and/or a non-exclusive licence for contributions from authors who are: i) UK Crown employees; ii) where BMJ has agreed a CC-BY licence shall apply, and/or iii) in accordance with the terms applicable for US Federal Government officers or employees acting as part of their official duties; on a worldwide, perpetual, irrevocable, royalty-free basis to BMJ Publishing Group Ltd ("BMJ") its licensees and where the relevant Journal is co-owned by BMJ to the co-owners of the Journal, to publish the Work in this journal and any other BMJ products and to exploit all rights, as set out in our licence.

The Submitting Author accepts and understands that any supply made under these terms is made by BMJ to the Submitting Author unless you are acting as an employee on behalf of your employer or a postgraduate student of an affiliated institution which is paying any applicable article publishing charge ("APC") for Open Access articles. Where the Submitting Author wishes to make the Work available on an Open Access basis (and intends to pay the relevant APC), the terms of reuse of such Open Access shall be governed by a Creative Commons licence – details of these licences and which Creative Commons licence will apply to this Work are set out in our licence referred to above.

Other than as permitted in any relevant BMJ Author's Self Archiving Policies, I confirm this Work has not been accepted for publication elsewhere, is not being considered for publication elsewhere and does not duplicate material already published. I confirm all authors consent to publication of this Work and authorise the granting of this licence.

For peer review only

Governance for injury care systems in Ghana, South Africa and Rwanda: development and pilot testing of an assessment tool

The Equi-Trauma Collaborative

Maria Lisa Odland,[1-3] Abdul-Malik Abdul-Latif,[1,4] Agnieszka Ignatowicz,[1] Abebe Bekele,[5-6] Kathryn Chu,[7-8] Anthony Howard,[9-10] Stephen Tabiri,[11-12] Jean Claude Byiringiro,[13-14] Justine I Davies[1,15,16]

1. Institute of Applied Health Research, University of Birmingham, Birmingham, U, K.
2. Department of Obstetrics and Gynecology, St. Olavs University Hospital, Trondheim, Norway
3. Malawi-Liverpool-Wellcome Trust Research Institute, Blantyre, Malawi
4. Volta Regional Health Directorate, Ghana Health Service, Ho, Ghana
5. University of Global Health Equity, Kigali, Rwanda
6. Department of Surgery, Addis Ababa University, Addis Ababa, Ethiopia
7. Centre for Global Surgery, Department of Global Health, Stellenbosch University, Cape Town, South Africa
8. Department of Surgery, University of Botswana, Gaborone, Botswana
9. Leeds Institute of Rheumatic and Musculoskeletal Medicine, School of Medicine, University of Leeds, Leeds, UK
10. Nuffield Department of Orthopaedics, Rheumatology and Musculoskeletal Sciences, National Institute of Health Research (NIHR) Biomedical Centre, University of Oxford, Headington, Oxford, UK
11. Ghana HUB of NIHR Global Surgery, Tamale, Ghana. Department of Public Health, Tamale Teaching Hospital, Tamale, Ghana
12. Department of Surgery, Tamale Teaching Hospital, Tamale, Ghana
13. University of Rwanda, Kigali, Rwanda
14. Department of Surgery, University Teaching Hospital of Kigali, Kigali, Rwanda
15. Centre for Global Surgery, Department of Global Health, Stellenbosch University, Cape Town, South Africa
16. Medical Research Council/Wits University Rural Public Health and Health Transitions Research Unit, Faculty of Health Sciences, School of Public Health, University of the Witwatersrand, Johannesburg, South Africa

Corresponding author:

Agnieszka Ignatowicz, a.m.ignatowicz@bham.ac.uk, University of Birmingham, Birmingham Clinical Trials Unit College of Medical and Dental Sciences, Birmingham B15 2TT, United Kingdom,+44 (0) 121 414 3344

Keywords: Injuries, Governance, Trauma, Health Policy, Public Health

Word count: 3926 excluding title page, abstrac references, figures and tables.

For peer review only

Abstract

Introduction

Injury is a leading cause of disability globally and responsible for more than 5 million deaths yearly, with 90% being in low- and middle-income countries. Still, little is done to monitor and evaluate healthcare governance for injury and trauma in this context.

Methods

Based on Siddiqi at al.'s framework for governance, we developed an online assessment tool for health system governance for injury with 37 questions covering health policy and implementation under ten overarching principles of Strategic vision, Participation and Consensus orientation, Rule of Law, Transparency, Responsiveness of Institutions, Equity, Effectiveness or Efficiency, Accountability, Ethics, and Intelligence/information. The tool was sent out to purposively selected stakeholders, including policymakers and injury care providers in Ghana, Rwanda and South Africa. Data were collected between October 2020 and February 2021. A literature reivew was also done to support the scoring. We derived scores using two methods - investigator scores and respondent scores.

Results

Rwanda had the highest overall investigator percentage score (70%), followed by South Africa (59%). Ghana had the lowest overall investigator score (48%). The overall results were similar for the respondent scores. Some areas such Participation and Consensus scored high in all three countries, whilst other areas such Transparency scored very low.

For peer review only

Conclusion

In this multi-country governance survey, we have shown that injury governance structures are limited in three Sub-Saharan African countries. This study provides insight into the governance of trauma systems in these three countries. It highlights areas that need to be prioritised to meet the increasing burden of trauma and injuries.

For peer review only

What is already known about this topic

- Injuries and trauma are major causes of mortality, especially in low and middle-income countries.
- There is a recognised need to invest in health services to provide trauma care. However, little is done to monitor and evaluate healthcare governance for trauma in low-and middle-income countries.

What does this study add

- We assessed the health systems governance for trauma care in three diverse Sub-Saharan African countries - Ghana, Rwanda and South Africa - to try to understand the foundations on which to build improved health systems for trauma.
- The application of our adapted tool revealed strengths and weaknesses in policies and governance of trauma care.

How can this study might affect research, policy and practice

- Assessing health systems governance for injury, as we did in this study, provides evidence that should not only stimulate more research in this area but also support advocacy efforts to advance trauma care systems.

For peer review only

Introduction

Injury is a leading cause of disability globally and responsible for more than 5 million deaths each year.[1] Mortality from injury account for more deaths than tuberculosis (TB), malaria and HIV combined,[1] and 90% of these deaths occur in low- and middle-income countries (LMICs).[2] Deaths from injury are predicted to become the leading cause of death by 2030.[3] Despite this increasing burden, only a few LMICs have well-defined trauma systems or trauma registries.[4]

The United Nations Development Program defines governance as the exercise of political, economic, and administrative authority in managing a country's affairs at all levels.[5] Governance has long been a critical factor that influences a country's economic growth, social advancement, and general development. It is recognised as especially important for advancing progress towards attaining the Sustainable Development Goals (SDGs) in LMICs.[6] Over recent years, there has been increasing interest in health systems governance (HSG) with the recognition that good governance leads to better health outcomes for individuals and populations.[6] The Lancet-University of Oslo Commission on Global Governance for Health in 2014 called for a cross-sectoral global action and platform for health governance to serve as a policy forum to allow the contribution of diverse stakeholders to frame issues, set agendas, and debate policies that affect health and health equity.[7] The World Health Organisation first introduced the term "stewardship" – a part of governance, in the year 2000, and called for strategic policy frameworks that would allow the incorporation of effective oversight, regulation, incentives, and accountability in health governance.[8]

HSG thus involves setting evidence-based shared strategic visions and objectives, in addition to making policies, legislation, and deploying resources to ensure the goals and objectives are achieved.[9] However, despite its importance in supporting the delivery of better services and

For peer review only

improved health outcomes, little is done to monitor and evaluate HSG in LMICs.[10-12] Additionally, literature is scarce on HSG around trauma care in LMICs.[13] Previous studies on HSG have primarily focused on general health systems functions and particularly on the role of government in governance and the involvement of communities.[14] Moreover, the sparse disease-specific literature that exists focuses on the governance of programs for immunisation,[15] TB control,[16] mental health care,[17-19] and achieving global HIV goals.[20]

Given the prevalence of injury and the recognised need to invest in health services to provide trauma care, good governance will be essential to ensure the care provided is of high quality and accessible to those who need it. As part of a larger project that identified barriers in access to quality care for people who have been injured in LMICs [21], we adapted a tool to access the HSG for trauma care in three diverse countries in Sub-Saharan Africa - Ghana, Rwanda and South Africa - in order to try to understand the foundations on which to build improved health systems for trauma.

Methods

Study setting

The study was conducted in three LMIC countries in sub-Sahara Africa: Ghana, Rwanda and South Africa which have vastly different development and health systems. Ghana is a lower middle-income country, an estimated population of 30.4 million people (2019), a life expectancy of 63.8 years, and a Gross National Income (GNI) per capita of $2,220.[22] While health service delivery in the country is largely provided by government, private health institutions also provide significant proportion of health services to the population.[23] The National Ambulance Service provides 24-hour pre-hospital care for accidents and emergencies as part of the care provided by the government.[23] It has been estimated that

For peer review only

7.56% of deaths and 7.24% of disability-adjusted life years (DALYs) in Ghana are due to trauma.[22 24]

Rwanda, a landlocked East African country of 12.6 million people, has a life expectancy of 68.7 years and GNI per capita of $830.[22] It is classified as a low-income country. Around 9% of all deaths and 10% *of* DALYs are due to trauma.[22] Following the near decimation of its health system by the 1994 genocide, the country has taken steps to strengthen it, giving autonomy to District Health Services to serve urban and rural zones.[25] It introduced the Community-Based Health Insurance (CBHI) system in 1999/2000 to provide health insurance to rural populations.[26] However, the health system is still challenged, and deficiencies exist in the provision of quality trauma care.[27]

South Africa is an upper-middle-income country, with a population of 68.6 million and a life expectancy of 63.8 years. Injuries are estimated to be responsible for 10% of death and 11% *of* DALYs.[22 24] South Africa has the third biggest economy in Africa and a GNI per capita of $6,040.[22] Most South Africans (84%) access health services through government clinics, whilst the more affluent people go to private hospitals.[28]

Data collection

Building on the framework and tool developed by Siddiqi et al.[6] for assessing the HSG in developing countries, we developed an assessment tool for injury/trauma HSG with 37 questions covering health policy and implementation using the ten overarching principles outlined by Siddiqi et al.: Strategic vision, Participation and Consensus orientation, Rule of Law, Transparency, Responsiveness of Institutions, Equity, Effectiveness or Efficiency, Accountability, Ethics, and Intelligence/information (Appendix 1 and Table 1). Adjustments to the original tool were made to tailor question to trauma; these were made based upon

For peer review only

discussion between the authors of this paper. The resultant tool was piloted for acceptability and comprehensibility before use. Data were collected over a five-month period from October 2020 to February 2021 by participants self-completing an online word or google form, based on their preference. Participants were requested to select a single response for each question and included a free text field for notes and provision of evidence to support their responses was encouraged. All responses were imported and analysed in Microsoft Excel.

An extensive review of documents from grey and published literature was also done to support the assessment, parricularly the scoring.

Survey respondent selection

Our aim was to recruit participants from health policy or senior leaders in trauma care provision in each country. Given that we expected potential participants to have sound knowledge of the policy and governance context for injury care in their countries, we aimed for a sample size of 5-8 respondents and contacted potential participants until at least the minimum number was achieved. A combination of purposive and snowball sampling was used to recruit respondents, with potential participants identified with the support of in-country senior researchers within injury care. Emails were sent to potential participants to request their participation in the study; for each invited participant, two further reminders were sent.

Grey literature search

We additionally conducted a search for and review of program documents, policies, annual reports, and standard operating procedures for each country. Searching was done through the websites of government organisations at the national and sub-national level, websites of

For peer review only

international organisations, and Google search engine. The search terms included the country name and trauma policy, trauma law, strategic plan for trauma, injury, injury work plan, injury policy, injury care, trauma care, injury guidelines, trauma guidelines, and combinations of these. There was no restriction on the year of publication.

Scoring

Scoring was done separately for each country. For each question, responses were awarded points and treated as binary categorical or ordinal (see appendices). We derived scores using two methods - investigator scores and respondent scores. The investigator scores considered the following to derive a final score for each question: results from respondents, respondents' professional roles, and the availability of evidence from policy documents and guidelines undertaken as a part of grey literature searches. Investigator scores were derived after discussions between the authors. Consideration of the respondent's professional roles depended on the question asked; more emphasis was given to responses from policymakers rather than trauma care providers for policy-related questions, and more given to trauma care providers where questions were related to service provision. So, for example, if a trauma care provider gave a score of 0, and the policymaker gave a score of 2 on a question related to policy, such as; *are there legal documents of injury care*?, the question would receive a final score of 2 as the policymaker was more likely to have up-to-date knowledge on this. If there was a policy document available to definitively answer a question, the literature took precedence over respondents. This process was done through discussions between two authors: AMAL and ML. Where there were disagreements, a third and fourth investigator served as an arbiter (AI and JD).

For peer review only - http://bmjopen.bmj.com/site/about/guidelines.xhtml

For the respondent scores, the mean score across respondents for each question was simply computed as the average score from the responses for each question. Given that the response rate for each country differed, the denominator (n) varied based on the number of responses: 11 for Ghana, 5 for Rwanda and 4 for South Africa.

Both investigator scores and the average respondent scores for each principle were calculated by dividing the achieved score in each country by the total score possible to achieve and multiplying it by a 100. Comparisons across countries are described at the level of the 10 Principles and overall.

For peer review only

Table 1. Applying Siddiqi et al. governance framework to trauma care systems in Ghana, Rwanda and South Africa. Questions for each governance principle are in table 3

S/N	Governance principle	Explanation of principles based upon from Siddiqi et al (2008)	Domain captured for trauma care	Maximum score for principle
**1**	Strategic vision	Through an understanding of the historical, cultural and social complexities of society, leaders have a strong sense of direction for the achievement of long and broad health and human development goals.	There is a detailed long term strategic plans to improve trauma care	12
**2**	Participation and consensus orientation	Everyone or interest groups or institutions acting on behalf of everyone should be given the chance to have a say in relation to decisions about health. This is built on the principle of freedom of association and speech as well as capacities to participate constructively. Good governance should be able to mediate between differing opinions among stakeholders on health, policies and procedures in order to reach a mutual understanding that is beneficial for all.	There is stakeholder participation and level of engagement in policy formulation and implementation for trauma	3
**3**	Rule of law	Legal frameworks or policies relating to human rights on health especially should be applied impartially	There is availability and enforcement of laws, guidelines, policies to support trauma care	6
**4**	Transparency	There should be free flow of information on all health matters. There should be enough information available to all to not only monitor but also understand health matters. Processes, institutions and information should be directly accessible to those concerned with them.	There is transparency on commitments to trauma and available information on indicators and other trauma related information for providers (district) involved in local trauma service provision	3

For peer review only

**5**	Responsiveness	Institutions and processes should promptly serve all stakeholders and ensure that their health and non-health needs are met without delays	Trauma systems are responsive to trauma care needs of the population	10
**6**	Equity and inclusiveness	Everyone should have the opportunity to improve or maintain their health and well being	There is equity in access to quality trauma care	8
**7**	Effectiveness and efficiency	Institutions and processes should maximize available resources to render best health care services according to population needs, as well as influence improved health outcomes	There is the existence of organisational capacity including human resource, communication processes to support quality trauma provision	6
**8**	Accountability	People put in positions of trust from government, the private sector and civil society organisations should be accountable to the public and institutional stakeholders. Accountability in this sense varies depending on the type of institution or organisation and whether or not decisions are for internal or external purposes	There is evidence of accountability between service providers and users in the provision of trauma care	3
**9**	Intelligence and information	Essentials for understanding of the health system to guide the implementation of good policies that are based on empirical data to influence the behaviour of different interest groups that support the strategic vison for health.	There is availability of tools and capacity to capture trauma care data	2
**10**	Ethics	Widely accepted principles of health care ethics; non-maleficence, beneficence and justice. This also includes ethics in health care research essential to safeguard the interest and rights of the patients.	There is enforcement of high ethical standards in trauma care provision and research	3
	**Maximum score**			**56**

For peer review only

Patient and public involvement

It was not appropriate or possible to involve patients or the public in the design, or conduct, or reporting, or dissemination plans of our research.

Results

Table 2 shows a breakdown of the number of respondents from each country and their employment role at the time of completing the survey. Respondents were made up of key officials employed directly by or advising Department or Ministry of Health, trauma care providers (some of whom were also involved in research), and government officials. Thirteen potential respondents were contacted from each country.

Table 2. Breakdown of respondents from each country and their characteristics

S/N	Country	No of potential participants contacted	No of respondents	No of policy respondents	No of trauma care providers
1	Ghana	13	11	3	8
2	Rwanda	13	5	3	2
3	South Africa	13	5	3	2

Appendix table 2 shows the investigator score for each country according to each question and percentage score for each principle and overall for each country. Some of the respondents provided evidence to support their answers such as policy documents and peer-reviewed papers.

Rwanda had the highest overall investigator percentage score (70%) followed by South Africa (59%). Ghana had the lowest overall investigator percentage score (48%) (Table 3). The overall results were similar for the respondent average percentage score, with Rwanda scoring 39.85

For peer review only

(71%) in total, South Africa 31.07 (56%) and Ghana 18.5 (33%) (Appendix Table 3 – with both percentage scores shown for comparison).

Table 3 Investigator score for each question and percentage score for each principle and overall for Rwanda, Ghana and South Africa respectively

Principle	One question out of many questions asked in this principles	Maximum score for questions	Rwanda score	Ghana score	South Africa score
**Strategic vision**	Is there specific mention of trauma in the national health plan or policy? Or are there specific national health policies around trauma care?	12	8 (67%)	4 (33%)	6 (50%)
**Participation and consensus**	What is the level of stakeholder engagement/community inparticipation at the national and provincial level in trauma policy and related interventions?	3	3 (100%)	3 (100%)	3 (100%)
**Rule of law**	Are there guidelines for accreditation of trauma care providers (doctors, nurses, etc) and are these enforced?	6	4 (67%)	5 (83%)	5 (83%)
**Transparency**	Are managers (District Directors of Health, Medical Superintendents of hospitals) evaluated on their health facility or facilities reaching specific targets for trauma care? And if so, are the results of these evaluations available and accessible?	3	3 (100%)	0 (0%)	0 (0%)
**Responsiveness of institutions**	Is there mandatory reporting of health facility trauma data and is this used to define the burden of injury at a national level?	10	8 (80%)	5 (50%)	5 (50%)

For peer review only

**Equity**	Are there national level financial schemes to ensure the poor who are injured do not have to pay out of pocket direct medical costs of trauma care?	8	3 (38%)	2 (25%)	3 (38%)
**Effectiveness and efficacy**	Is there a national trauma registry (information management for trauma care)? Is it used? In both private and public?	6	5 (83%)	4 (67%)	4 (67%)
**Accountability**	Are there mechanisms to report failing trauma services to policy makers or regulatory authorities?	3	1 (33.3%)	1 (33.3%)	3 (100%)
**Intelligence/information**	Do staff providing trauma services understand what data needs to be captured and do they have the right data capturing tools to enable them to do this?	3	1 (33%)	1 (33%)	1 (33%)
**Ethics**	Are there any standard operating procedures in place to ensure quality and ethical trauma care for injured people?	3	3 (100%)	2 (67%)	3 (100%)
**Overall Total (% maximum overall score)**		**56**	**39 (70%)**	**27 (48%)**	**33 (59%)**

Considering the investigator scores, Rwanda had the highest scores for each Principle except for Equity. Participation and consensus orientation in particular had a very high score in Rwanda (100%), whilst the other scores were between 70-80%, apart from Strategic vision (66.7%) and Equity (37.5%). Like Rwanda, South Africa also had high investigator-weighted scores overall, but had low scores for Strategic vision (50.0%), Equity (37.5%), and Intelligence and Information (50.0%). For Transparency, South Africa had a score of 0%. On the other

For peer review only

hand, South Africa had high scores for Participation and consensus orientation (100%), Rule of Law (83.3%), Accountability (100%) and Ethics (100%). Responsiveness of institutions (50.0%) and Effectiveness and efficiency (66.7% and 66.7%) received medium high scores. Ghana's highest scores were for the Principles of Rule of Law (83.30%), Effectiveness and Efficiency (66.70%) and Ethics (66.70%). However, the scores were low for the other Principles, especially Strategic Vision (33.30%), Transparency (0%), Equity (25%) and Accountability (33.30%). This gave Ghana the overall lowest score in the Governance Assessment for Trauma with an investigator score of 48.20% (see table 3). The only Principle that received a 100% investigator score in all the countries was Participation and consensus orientation.

Discrepancies between investigator scores and average respondent scores were mostly seen in Ghana, where the overall scores were 33.0% versus 48.20%, respectively, and there were fairly large discrepancies for almost all the Principles except for Equity (17.5% versus 25.%), Effectiveness and efficiency (54.7% versus 66.70%), and Accountability (28.70% versus 33.30%) (Appendix 2) (Appendix table 3). The average respondent and investigator percentage scores for each Principle were more similar for the other two countries. In Rwanda, the overall average respondent percentage score was 71.2%, and the average percentage investigator score was 69.6%; most of the individual Principles had similar respondent percentage scores except for Transparency (60.0% versus 100.0%), Accountability (70.0% versus 33.3%) and Intelligence and Information (80.0% versus 50.0%). In South Africa, the overall average respondent percentage score was 55.5%, and the overall investigator percentage score was 58.9%. Similar to Rwanda, the individual Principle scores were more or

less similar except for those of Strategic vision (38.9% versus 50.0%), Accountability (89.0% versus 100.0%), Intelligence/information (37.5% and 50.0%) and Ethics (89.0% versus 100%).

Discussion

To the best of our knowledge, this is the first study that has adapted assessed governance for trauma health systems across multiple countries. The application of our adapted tool revealed strengths and weaknesses in policies and governance of trauma care in Ghana, South Africa and Rwanda. Rwanda achieved fairly high scores (70%), followed by South Africa (59%), and Ghana had the lowest score (40%). However, considering the massive burden of injuries and trauma in these countries, our results suggest that even in the higher performing countries there is room for improvement. Whereas the gap between the burden of disease and available governance systems and structures was especially seen in Ghana, the focus on other conditions in Ghana, such as the free maternal health care policy - which has been vital in ensuring access to health care for women and children – shows what is possible to achieve [29].

Rwanda scored relatively high in our survey. Reasons for this could be that having successfully achieved the MDGs, Rwanda has committed to reducing morbidity and mortality due to injuries,[8 30] including developing policies, training healthcare providers, investing in data-collection, and hosting first national symposium on trauma and injuries in 2019.[31] Hence there has been a focus on improving health systems to care for patients with injuries in the last few years. There is still high mortality and morbidity from injuries in the country, but interventions following recent policies and prioritisation of trauma care coupled with efforts to prevent injuries – for example, the recent introduction of speed cameras in urban areas - is likely to improve the situation in the years to come.

For peer review only

Given the level of development – being the only upper-middle income country in our study, it is surprising that South Africa had mediocre percentage scores of around 50%. Many LMICs have a high burden of injuries and trauma, but South Africa has a relatively large burden of homicide, violence and stabbings[32] in addition to other common injuries such as road traffic accidents and burns. Even though there are programs, services, and ongoing research on this topic, there has been an absence of government stewardship and leadership.[32] In South Africa, in particular, prevention of violence and injury should be a strategic priority for government programmes and policies. There are valuable lessons that South Africa can learn from its own excellent governance structures for HIV care.[33]

Overall our results emphasise that more efforts are needed to strengthen overall governance for injury care, considering how crucial governance is to achieve Universal Health Coverage (UCH).[34] Finance cannot be neglected in this process, however it is also critical to focus on the Principles that were particularly weak in this study (transparency, accountability and intelligence/information), to improve the effectiveness of the health sector.[34] We found that not only was the availability of financial commitments to trauma care in the public sector a challenge, but also accountability in relation to adequate data generation and the correction of trauma care underperformance. Whereas, WHO has developed a trauma registry for LMIC settings which can be tailored to individual country needs, uptake of this at national levels is lacking and use of data collected for health service quality improvement is under-developed. Rwanda is the only country in our study that uses the WHO-based trauma registry, and this is only used in 5 hospitals and without an active quality improvement program – although there are plans to develop this.[30]

For peer review only

Another thing that was evident in our findings was the difference between the investigator scores and the respondent scores. Overall, Rwanda had the highest score regardless of the scoring system used, and the overall investigator score, and respondent score were similar. However, in the other two countries the respondent score was lower than the investigator score, especially in Ghana, which also had the lowest scores altogether. The difference between the respondent and investigator score suggests that many respondents are not aware of relevant policies/governance structures for trauma in their respective countries. Awareness of these is the first step to using them in order to improve injury and trauma care in the respective countries; the quality of governance is associated with health systems performance;[35] however, policies are of no use if the people in charge of implementing them are not aware of them. According to our survey, this is mostly an issue in Ghana, but also somewhat in South Africa.

This study also revealed some interesting findings in relation to "Participation and consensus orientation" as it was the only principle where all three countries scored 100%. More involvement of stakeholders may improve service delivery and reduce barriers to accessing quality care for injuries after trauma. But, this isn't necessarily the case, as seen in Nigeria, where an increased involvement of stakeholders in the formulation and implementation of TB policies[16] did not necessarily result in good TB control in the community and the health services in the country.

It is likely that multiple components of governance need to be in place – in combination with the awareness of these – for the improvement of healthcare systems. For example, in Ethiopia improved health system governance was expected to impact critically on scaling up mental health care within primary care facilities.[17] The presence of high-level government support

For peer review only - http://bmjopen.bmj.com/site/about/guidelines.xhtml

was thought to be a strength along with a National Mental Health Strategy. But unfortunately, there was still a very low baseline awareness of mental health care planning and a lack of leadership and coordination of mental health planning at the national and district level. Indeed, a qualitative study using Saddiqi's framework for mental health governance in South Africa found that facilitating factors to implementing integrated mental health care were using task-sharing models, establishment of district mental health teams to facilitate the development, and implementation of mental health care plans. The challenges were weak managerial and planning capacity to develop health care at the provincial and district level. All of which speak to the need for knowledge and implementation of governance structures for the improvement of healthcare. Hence to strengthen health care delivery there is a critical need to strengthen leadership and coordination, and implementation at all levels; national, regional, district, and down to individual healthcare facilities. There are valuable lessons from these other disease areas that can be used for using governance structures for improving trauma care systems.

In this survey, we managed to obtain responses from a range of professionals working with trauma care in three different countries in Sub-Saharan Africa. However, a major limitation is that we only had five participants in two of the countries (Rwanda and South Africa) and found soliciting involvement of respondents difficult, despite having researchers with links to policy makers leading the study in each country. The low numbers of respondents could have introduced selection bias. We tried to overcome this bias using an investigator score. However, we may have found different results if we had achieved greater numbers of participants from each country. Nevertheless, our results have face validity, considering that injury care has been an area of focus in Rwanda[31], and Rwanda scored highest in our

For peer review only - http://bmjopen.bmj.com/site/about/guidelines.xhtml

governance survey. The investigator scores also had its limitations. If there was no available evidence the investigators had to weigh the replies from the respondents according to their background which involved making assumptions about the respondents knowledge of the subject, which may have been false. We did our outmost to make sure the investigator scores were correct by checking the grey literature and available information. Our scoring system has not been validated and we cannot be certain that the scores were always reflective of the true trauma systems governance of that country, or that one country is doing better than the other.

Nevertheless, our study is novel in looking at governance assessment for injuries in LMICs. This survey tool provides useful insight in the governance of trauma systems in three LMIC countries with different development status and provides evidence that governance systems for trauma need to be improved in certain areas in order to face the increasing burden of injuries in LMICs in the years to come.

Conclusions

In this multi-country governance survey, we have shown that the governance structures for trauma is limited in three different countries in Sub-Saharan Africa; Ghana, Rwanda and South Africa. Some areas, such Participation and consensus, scored high in all three countries whilst other areas such Transparency scored very low. This study provides insight into the governance of trauma systems in these three countries and highlights areas that need to be prioritised in the years to come in order to meet the increasing burden of trauma and injuries. Assessment of the health systems governance for trauma, as we did in this study, provides

evidence that should not only stimulate more research in this area but also support advocacy efforts to advance trauma care systems.

For peer review only

References

1. World Health Organisation. Injuries and Violence the Facts 2014 [Available from: https://apps.who.int/iris/rest/bitstreams/668802/retrieve.
2. Gosselin RA, Spiegel DA, Coughlin R, et al. Injuries: the neglected burden in developing countries. *Bull World Health Organ* 2009;87(4):246-46a. doi: 10.2471/blt.08.052290 [published Online First: 2009/06/25]
3. Mathers CD, Loncar D. Projections of global mortality and burden of disease from 2002 to 2030. *PLoS Med* 2006;3(11):e442. doi: 10.1371/journal.pmed.0030442 [published Online First: 2006/11/30]
4. Whitaker J, O'Donohoe N, Denning M, et al. Assessing trauma care systems in low-income and middle-income countries: a systematic review and evidence synthesis mapping the Three Delays framework to injury health system assessments. *BMJ Glob Health* 2021;6(5) doi: 10.1136/bmjgh-2020-004324 [published Online First: 2021/05/13]
5. United Nations. Human Development Report 1997 [Available from: http://hdr.undp.org/sites/default/files/reports/258/hdr_1997_en_complete_nostats.pdf.
6. Siddiqi S, Masud TI, Nishtar S, et al. Framework for assessing governance of the health system in developing countries: gateway to good governance. *Health Policy* 2009;90(1):13-25. doi: 10.1016/j.healthpol.2008.08.005 [published Online First: 2008/10/08]
7. Ottersen OP, Dasgupta J, Blouin C, et al. The political origins of health inequity: prospects for change. *Lancet* 2014;383(9917):630-67. doi: 10.1016/S0140-6736(13)62407-1 [published Online First: 2014/02/15]
8. World Health Organisation. Everybody's business: Strengthening health systems to improve health outcomes 2007 [Available from: https://www.who.int/healthsystems/strategy/everybodys_business.pdf.
9. Lewis M. and Petterson G. Governance in Health Care Delivery 2009 [Available from: https://poseidon01.ssrn.com/delivery.php?ID=538097071083084007079107106066114005010087014021019030101052032018059029044024014036025095087006090007037092008070123064093068026009089080094025102105080027103111123030016094008003098115064&EXT=pdf&INDEX=TRUEw.
10. Mugisha J, Ssebunnya J, Kigozi FN. Towards understanding governance issues in integration of mental health into primary health care in Uganda. *Int J Ment Health Syst* 2016;10:25. doi: 10.1186/s13033-016-0057-7 [published Online First: 2016/03/26]
11. Bossert TJ. Health systems. *Health Policy Plan* 2012;27(1):8-10. doi: 10.1093/heapol/czr008 [published Online First: 2011/02/19]
12. Cole E. The national major trauma system within the United Kingdom: inclusive regionalized networks of care. *Emergency and Critical Care Medicine* 2022;2(2):76-79. doi: 10.1097/ec9.0000000000000040
13. Reynolds TA, Stewart B, Drewett I, et al. The Impact of Trauma Care Systems in Low- and Middle-Income Countries. *Annu Rev Public Health* 2017;38:507-32. doi: 10.1146/annurev-publhealth-032315-021412 [published Online First: 2017/01/27]
14. Rosato M, Laverack G, Grabman LH, et al. Community participation: lessons for maternal, newborn, and child health. *Lancet* 2008;372(9642):962-71. doi: 10.1016/S0140-6736(08)61406-3 [published Online First: 2008/09/16]
15. Grundy J. Country-level governance of global health initiatives: an evaluation of immunization coordination mechanisms in five countries of Asia. *Health Policy Plan* 2010;25(3):186-96. doi: 10.1093/heapol/czp047 [published Online First: 2009/11/21]
16. Ogbuabor DC, Onwujekwe OE. Governance of tuberculosis control programme in Nigeria. *Infect Dis Poverty* 2019;8(1):45. doi: 10.1186/s40249-019-0556-2 [published Online First: 2019/06/18]

17. Hanlon C, Eshetu T, Alemayehu D, et al. Health system governance to support scale up of mental health care in Ethiopia: a qualitative study. *Int J Ment Health Syst* 2017;11:38. doi: 10.1186/s13033-017-0144-4 [published Online First: 2017/06/13]
18. Petersen I, Marais D, Abdulmalik J, et al. Strengthening mental health system governance in six low- and middle-income countries in Africa and South Asia: challenges, needs and potential strategies. *Health Policy Plan* 2017;32(5):699-709. doi: 10.1093/heapol/czx014 [published Online First: 2017/04/04]
19. Marais DL, Petersen I. Health system governance to support integrated mental health care in South Africa: challenges and opportunities. *Int J Ment Health Syst* 2015;9:14. doi: 10.1186/s13033-015-0004-z [published Online First: 2015/03/26]
20. Khalid H, Fox AM. Political and Governance Challenges to Achieving Global HIV Goals with Injecting Drug Users: The Case of Pakistan. *Int J Health Policy Manag* 2019;8(5):261-71. doi: 10.15171/ijhpm.2018.131 [published Online First: 2019/06/18]
21. Odland ML, Abdul-Latif A-M, Ignatowicz A, et al. Equitable access to quality trauma systems in low-income and middle-income countries: assessing gaps and developing priorities in Ghana, Rwanda and South Africa. *BMJ Global Health* 2022;7(4):e008256. doi: 10.1136/bmjgh-2021-008256
22. The World Bank. World Bank Open Data 2021 [Available from: https://data.worldbank.org/.
23. Ghana Health Service Ministry of Health. Ghana Health Service 2014 Annual Report 2014 [Available from: https://www.ghanahealthservice.org/downloads/Ghana_Health_Service_2014_Annual_Report.pdf.
24. Institute for Health Metrics and Evaluation. Country profiles [Available from: http://www.healthdata.org/results/country-profiles.
25. The DHS Program. Rwanda: Standard DHS, 2019-20 2020 [
26. Nyandekwe M, Nzayirambaho M, Kakoma JB. Universal health insurance in Rwanda: major challenges and solutions for financial sustainability case study of Rwanda community-based health insurance part I. *Pan Afr Med J* 2020;37:55. doi: 10.11604/pamj.2020.37.55.20376 [published Online First: 2020/11/20]
27. Ntakiyiruta G, Wong EG, Rousseau MC, et al. Trauma care and referral patterns in Rwanda: implications for trauma system development. *Can J Surg* 2016;59(1):35-41. doi: 10.1503/cjs.008115 [published Online First: 2016/01/27]
28. Mahlahti P. and Dlamini J. MINIMUM DATA SETS FOR HUMAN RESOURCES FOR HEALTH AND THE SURGICAL WORKFORCE IN SOUTH AFRICA'S HEALTH SYSTEM 2015 [Available from: https://www.who.int/workforcealliance/031616south_africa_case_studiesweb.pdf.
29. Adu J, Mulay S, Owusu MF. Reducing maternal and child mortality in rural Ghana. *Pan Afr Med J* 2021;39:263. doi: 10.11604/pamj.2021.39.263.30593 [published Online First: 20210824]
30. Jayaraman S, Ntirenganya F, Nkeshimana M, et al. Building Trauma and EMS Systems Capacity in Rwanda: Lessons and Recommendations. *Ann Glob Health* 2021;87(1):104. doi: 10.5334/aogh.3324 [published Online First: 20211026]
31. Rosenberg A, Ntirenganya F, Bagahirwa I, et al. First Rwanda National Trauma Symposium 2019: Challenges and priorities. *J Glob Health* 2020;10(1):010201. doi: 10.7189/jogh.10.010201 [published Online First: 2020/04/08]
32. Seedat M, Van Niekerk A, Jewkes R, et al. Violence and injuries in South Africa: prioritising an agenda for prevention. *Lancet* 2009;374(9694):1011-22. doi: 10.1016/s0140-6736(09)60948-x [published Online First: 20090824]
33. Lodge T. The politics of HIV/AIDS in South Africa: government action and public response. *Third World Quarterly* 2015;36(8):1570-91. doi: 10.1080/01436597.2015.1037387
34. Fryatt R, Bennett S, Soucat A. Health sector governance: should we be investing more? *BMJ Glob Health* 2017;2(2):e000343. doi: 10.1136/bmjgh-2017-000343 [published Online First: 2017/12/12]

For peer review only - http://bmjopen.bmj.com/site/about/guidelines.xhtml

35. Olafsdottir AE, Reidpath DD, Pokhrel S, et al. Health systems performance in sub-Saharan Africa: governance, outcome and equity. *BMC Public Health* 2011;11:237. doi: 10.1186/1471-2458-11-237 [published Online First: 2011/04/19]

For peer review only

Appendix table 1. Copy of the governance survey

Principle	Questions	Responses (please circle one response for each question)	Maximum score
**Strategic vision**	Is there a specific mention of trauma in the national health plan or policy? Or are there specific national health policies around trauma	Don't know<br>No (0)<br>Yes (1)	**1**
	Is there a national trauma strategy?	Don't know<br>No (0)<br>Yes (1)	1
	What percentage of the country's health budget goes to trauma care?	Don't know<br><10% ((0)<br>10-20% (1)<br>>20% (2)	2
	Is public sector trauma care provided free of charge	Don't know<br>No-not at all (0)<br>Partly (1)<br>Yes-fully (2)	2
	Is there a national insurance scheme that covers trauma care	Don't know<br>No-not at all (0)<br>Partly (1)<br>Yes-fully (2)	2
	Is there a department within the National Ministry of Health dedicated for trauma?	Don't know<br>No department for trauma at all (0)<br>Department for trauma included in another department (1)<br>Stand-alone department for trauma (2)	2
	If the National health plan mentions trauma, or if there are national health policies for trauma care, are there specific objectives	Don't know<br>No (0)	1

For peer review only

	relating to trauma and timelines to achieve them?	Yes (1)	
	If you have answered yes to question 7, have any of those specific trauma objectives been implemented?	Don't know<br>No (0)<br>Yes (1)	1
Scores			12
**Participation and consensus orientation**	Are stakeholders (e.g: NGOs, private companies) involved in health policy formulation for trauma?	Don't know<br>No organisation is involved (0)<br>1-3 organisations are involved (1)<br>> 3 organisations are involved (2)	2
	What is the level of stakeholder engagement/community participation at the national and provincial level in trauma policy and related interventions?	Don't know<br>No involvement at all (0)<br>These groups are involved (1)	1
Scores			3
**Rule of law**	Are there guidelines for accreditation of trauma care providers (doctors, nurses, etc) and are these enforced?	Don't know<br>No guidelines present (0)<br>Guidelines present are not enforced (1)<br>Guidelines present and enforced (2)	2
	Are there laws to enforce a duty of care by hospitals to treat uninsured trauma patients?	Don't know<br>No laws (0)<br>Laws present are not enforced (1)<br>Laws present and enforced (2)	2
	Are there laws to protect against trauma? Example Seat belt laws?	Don't know<br>No laws (0)<br>Laws present are not enforced (1)	2

For peer review only

		Laws present and enforced (2)	
Scores			6
**Transparency**	Is information readily available on financial commitments or allocated budget to trauma care in the public sector at either national or provincial level?	Don't know<br>Don't know<br>No (0)<br>Yes (1)	1
	Are managers (District Directors of Health, Medical Superintendents of hospitals) evaluated on their health facility or facilities reaching specific targets for trauma care? And if so, are the results of these evaluations available and accessible?	Don't know<br>No trauma related criteria for assessment (0)<br>Trauma related criteria for assessment is used, but not available (1)<br>Trauma related criteria for assessment is used and available (2)	2
Scores			3
**Responsiveness of institutions**	Is there mandatory reporting of health facility trauma data and is this used to define the burden of injury at a national level?	Don't know<br>No (1)<br>Some data (1)<br>Adequate/complete data (2)	2
	Are these data used to inform national or provincial policy?	Don't know<br>No (0)<br>Yes (1)	1
	Are data on clinical post-injury outcomes captured routinely by health facilities?	Don't know<br>No (0)<br>Yes partially captured (1)<br>Yes adequately captured (2)	2

For peer review only

	Are data on post -injury care patient satisfaction routinely captured?	Don't know<br>No (0)<br>Yes partially captured (1)<br>Yes adequately captured (2)	2
	Are these trauma data used in planning services?	Don't know<br>No (0)<br>Yes (1)	1
	What is the level of responsiveness of the health system to non-medical needs of injured people (social needs, mental health needs etc) ?	Don't know<br>Health services cater to or assess only medical needs (1)<br>Health services consider other outcomes relevant to patients, for example their opinions on services provided (2)	2
Scores			10
**Equity**	Are there national level financial schemes to ensure the poor who are injured do not have to pay out of pocket direct medical costs of trauma care?	Don't know<br>No scheme (0)<br>Partial scheme (1)<br>All people are covered (2)	2
	Are there health policies in place to address inequality in access to care for trauma?	Don't know<br>No policies in place (0)<br>Policies in place for general health equity (1)<br>Policies in place specifically for trauma (2)	2
	Are there data which show whether access is equitable or not? And do those data show that access is equitable?	Don't know<br>No data available (0)<br>Data available (1)<br>Data available and show that access is equitable (2)	2

For peer review only

	Is the allocation of trauma care staff to districts and hospitals appropriate and based on needs? Do districts in urban centres get more staff than districts in remote areas?	Don't know<br>Less than 2:1 (1)<br>More than 2:1 (0)	1
	Is there a mechanism to equitably distribute the budget for health or trauma care?	Don't know<br>No (0)<br>Yes (1)	1
Scores			8
**Effectiveness and efficiency**	Is there a national trauma registry (information management for trauma care)? Is it used? In both private and public?	Don't know<br>No (0)<br>Yes (1)	1
	Are there national or provincial guidelines for in -service training of staff on trauma care?	Don't know<br>No (0)<br>Yes (1)	1
	Is there a pre-hospital referral system (e.g: ambulance service)?	Don't know<br>No (0)<br>Yes (1)	1
	If pre-hospital referral systems are available, what has been the experience of patients regarding pre hospital referrals?	Don't know<br>Poor (0)<br>Fair (1)<br>Good (2)	2
	Are local transport operators (e.g: taxis) involved in transporting people to hospital?	Don't know<br>No (0)<br>Yes (1)	1
Scores			6
**Accountability**	Are there mechanisms to report failing trauma services to policy makers or regulatory authorities	Don't know<br>No (0)<br>Yes (1)	1
	Are there any mechanisms of correcting under performance of trauma services?	Don't know<br>No mechanisms (0)	2

For peer review only

		Mechanisms of reporting but no onwards mechanisms to improve quality (1)<br><br>Mechanisms of reporting and mechanisms to improve quality (2)	
Scores			3
**Intelligence/Information**	Do staff providing trauma services understand what data needs to be captured and do they have the right data capturing tools to enable them do this?	Don't know<br><br>No understanding and no tools (0)<br><br>Understanding but no or limited tools (1)<br><br>Understanding and useful tools (2)	2
Scores			2
**Ethics**	Is there any policy available for regulating trauma related research?	Don't know<br><br>No (0)<br><br>Yes (1)	1
	Are there any standard operating procedures in place to ensure quality and ethical trauma care for injured people?	Don't know<br><br>No (0)<br><br>Yes (1)	1
	Are there mechanisms in place in institutes for enforcing high ethical standards in the treatment of trauma patients	Don't know<br><br>No (0)<br><br>Yes (1)	1
Scores			3
Overall maximum score			56

For peer review only

Appendix 2 Investigator score for each question and percentage score for each principle and overall for Rwanda, Ghana and South Africa respectively

Principle	Questions	Maximum score for question	Rwanda score	Ghana score	South Africa score
**Strategic vision**	Is there specific mention of trauma in the national health plan or policy? Or are there specific national health policies around trauma care?	1	1	1	1
	Is there a national trauma strategy?	1	1	0	0
	What percentage of the country's public health budget goes to trauma care?	2	0	0	2
	Is public sector trauma care provided free of charge?	2	1	1	1
	Is there a national insurance scheme that covers trauma care?	2	2	1	1
	Is there a department within the National Ministry of Health dedicated for trauma?	2	1	1	1
	If the National health plan mentions trauma, or if there are national health policies for trauma care, are there specific objectives relating to trauma and timelines to achieve them?	1	1	0	0
	If you have answered yes to question 7, have any of those specific trauma objectives been implemented?	1	1	0	0
**Score for Principle (% of total possible)**		**12**	**8 (67%)**	**4 (33%)**	**6 (50%)**
**Participation and consensus**	Are stakeholders (e.g: NGOs, private companies) involved in	2	2	2	2

For peer review only

	What is the level of stakeholder engagement/community participation at the national and	1	1	1	1
**Score for Principle (% of total possible)**		**3**	**3 (100%)**	**3 (100%)**	**3 (100%)**
**Rule of law**	Are there guidelines for accreditation of trauma care	2	2	2	1
	Are there laws to enforce a duty of care by hospitals to treat uninsured	2	0	2	2
	Are there laws to protect against trauma? Example Seat belt laws?	2	2	1	2
**Score for Principle (% of total possible)**		**6**	**4 (67%)**	**5 (83%)**	**5 (83%)**
**Transparency**	Is information readily available on financial commitments or allocated budget to trauma care in the public sector at either national or provincial level?	1	1	0	0
	Are managers (District Directors of Health, Medical Superintendents of hospitals) evaluated on their health facility or facilities reaching specific targets for trauma care? And if so, are the results of these evaluations available and accessible?	2	2	0	0
**Score for Principle (% of total possible)**		**3**	**3 (100%)**	**0 (0%)**	**0 (0%)**
**Responsiveness of institutions**	Is there mandatory reporting of health facility trauma data and is	2	2	1	1
	Are these data used to inform national or provincial policy?	1	1	1	1
	Are data on clinical post-injury outcomes captured routinely by	2	2	1	0
	Are data on post-injury care patient satisfaction routinely captured?	2	0	0	0
	Are these trauma data used in planning services?	1	1	1	1
	What is the level of responsiveness of the health system to non-medical needs of injured people (social needs, mental health needs etc)?	2	2	1	2

For peer review only

**Score for Principle (% of total possible)**		**10**	**8 (80%)**	**5 (50%)**	**5 (50%)**
**Equity**	Are there national level financial schemes to ensure the poor who are injured do not have to pay out	2	2	1	2
	Are there health policies in place to address inequality in access to care for trauma?	2	1	1	1
	Are there data which show whether access is equitable or not? And do those data show that access is equitable?	2	0	0	0
	Is the allocation of trauma care staff to districts and hospitals appropriate and based on needs? Do districts in urban centres get more staff than districts in remote areas?	1	0	0	0
	Is there a mechanism to equitably distribute the budget for health or trauma care?	1	0	0	0
**Score for Principle (% of total possible)**		**8**	**3 (38%)**	**2 (25%)**	**3 (38%)**
**Effectiveness and efficacy**	Is there a national trauma registry (information management for trauma care)? Is it used? In both private and public?	1	1	0	0
	Are there national or provincial guidelines for in-service training of staff on trauma care?	1	1	1	1
	Is there a pre-hospital referral system (e.g: ambulance service)?	1	1	1	1
	If pre-hospital referral systems are available, what has been the experience of patients regarding pre-hospital referrals?	2	1	1	2
	Are local transport operators (e.g: taxis) involved in transporting	1	1	1	0
**Score for Principle (% of total possible)**		**6**	**5 (83%)**	**4 (67%)**	**4 (67%)**

For peer review only

**Accountability**	Are there mechanisms to report failing trauma services to policy	1	0	1	1
	Are there any mechanisms of correcting under performance of	2	1	0	2
**Score for Principle (% of total possible)**		**3**	**1 (33.3%)**	**1 (33.3%)**	**3 (100%)**
**Intelligence/information**	Do staff providing trauma services understand what data needs to be captured and do they have the right	3	1	1	1
**Score for Principle (% of total possible)**		**3**	**1 (33%)**	**1 (33%)**	**1 (33%)**
**Ethics**	Is there any policy available for regulating trauma related research?	1	1	1	1
	Are there any standard operating procedures in place to ensure quality and ethical trauma care for injured people?	1	1	0	1
	Are there mechanisms in place in institutes for enforcing high ethical standards in the treatment of trauma patients?	1	1	1	1
**Score for Principle (% of total possible)**		**3**	**3 (100%)**	**2 (67%)**	**3 (100%)**
**Overall Total (% maximum overall score)**		**56**	**39 (70%)**	**27 (48%)**	**33 (59%)**

For peer review only

Appendix table 3: Summary results by principle for each country individually including average score, investigator score, maximum score possible for each question and achieved percentage score (average score and investigator score).

		Rwanda				Ghana				South Africa			
**Principle**	**Maximum scores**	**Respondent scores (average [%])**	**Investigator scores (number [%])**	**% achieved (average scores)**	**% achieved (Investigator scores)**	**Respondent scores (average [%])**	**Investigator scores (number [%])**	**% achieved (average scores)**	**% achieved (Investigator scores)**	**Respondent scores (average [%])**	**Investigator scores (number [%])**	**% achieved (average scores)**	**% achieved (Investigator scores)**
Strategic vision	12	8.05	8	67.1%	66.7%	2.07	4	17.30%	33.30%	4.67	6	38.9%	50.0%
Participation and consensus orientation	3	3	3	100.0%	100.0%	1.4	3	46.70%	100%	3	3	100.0%	100.0%
Rule of law	6	4.75	4	79.2%	66.7%	3.16	5	63.20%	83.30%	4.83	5	80.5%	83.3%
Transparency	3	1.8	3	60.0%	100.0%	0.49	0	16.30%	0%	0	0	0.0%	0.0%
Responsiveness of institutions	10	7.65	8	76.5%	80.0%	3.54	5	35.40%	50%	5.25	5	52.5%	50.0%
Equity	8	2.95	3	36.9%	37.5%	1.4	2	17.50%	25%	3.23	3	40.4%	37.5%
Effectiveness and efficiency	6	5.35	5	89.2%	83.3%	3.28	4	54.70%	66.70%	4	4	66.7%	66.7%
Accountability	3	2.1	1	70.0%	33.3%	0.86	1	28.70%	33.30%	2.67	3	89.0%	100.0%
Intelligence/information	2	1.6	1	80.0%	50.0%	0.73	1	36.50%	50%	0.75	1	37.5%	50.0%
Ethics	3	2.6	3	86.7%	100.0%	1.57	2	52.30%	66.70%	2.67	3	89.0%	100.0%
**Overall score**	**56**	**39.85**	**39**	**71.2%**	**69.6%**	**18.5**	**27**	**33.00%**	**48.20%**	**31.07**	**33**	**55.5%**	**58.9%**

Governance for injury care systems in Ghana, South Africa and Rwanda: development and pilot testing of an assessment tool

Journal:	*BMJ Open*
Manuscript ID	bmjopen-2023-074088.R1
Article Type:	Original research
Date Submitted by the Author:	10-Jul-2023
Complete List of Authors:	The Equi-Trauma, Collaborative; University of Birmingham Institute of Applied Health Research<br>Odland, Maria Lisa; University of Birmingham, Institute of Applied Health Research; St Olavs Hospital Trondheim University Hospital, Department of Obstetrics and Gyneacology<br>Abdul-Latif , Abdul-Malik ; Ghana Health Service, Volta Regional Health Directorate<br>Ignatowicz, Agnieszka ; University of Birmingham Institute of Applied Health Research<br>Bekele, Abebe; University of Global Health Equity<br>Chu, Kathryn; Stellenbosch University Faculty of Medicine and Health Sciences, Centre for Global Surgery, Department of Global Health; University of Botswana, Department of Surgery<br>Howard, Anthony; University of Leeds Leeds Institute of Rheumatic and Musculoskeletal Medicine; University of Oxford, Nuffield Department of Orthopaedics, Rheumatology and Musculoskeletal Sciences, National Institute of Health Research (NIHR) Biomedical Centre<br>Stephen, Tabiri; Ghana Hub of NIHR Global Surgery; Tamale Teaching Hospital, Department of Surgery<br>BYIRINGIRO, JEAN CLAUDE; University of Rwanda College of Medicine and Health Sciences; University Teaching Hospital of Kigali, Department of Surgery<br>Davies, Justine ; University of Birmingham, Institute of Applied Health Research; Stellenbosch University Faculty of Medicine and Health Sciences, Centre for Global Surgery, Department of Global Health
**Primary Subject Heading**:	Global health
Secondary Subject Heading:	Emergency medicine, Global health, Health services research, Health policy
Keywords:	Health policy < HEALTH SERVICES ADMINISTRATION & MANAGEMENT, Trauma management < ORTHOPAEDIC & TRAUMA SURGERY, ACCIDENT & EMERGENCY MEDICINE, Clinical governance < HEALTH SERVICES ADMINISTRATION & MANAGEMENT

SCHOLARONE™
Manuscripts

I, the Submitting Author has the right to grant and does grant on behalf of all authors of the Work (as defined in the below author licence), an exclusive licence and/or a non-exclusive licence for contributions from authors who are: i) UK Crown employees; ii) where BMJ has agreed a CC-BY licence shall apply, and/or iii) in accordance with the terms applicable for US Federal Government officers or employees acting as part of their official duties; on a worldwide, perpetual, irrevocable, royalty-free basis to BMJ Publishing Group Ltd ("BMJ") its licensees and where the relevant Journal is co-owned by BMJ to the co-owners of the Journal, to publish the Work in this journal and any other BMJ products and to exploit all rights, as set out in our licence.

The Submitting Author accepts and understands that any supply made under these terms is made by BMJ to the Submitting Author unless you are acting as an employee on behalf of your employer or a postgraduate student of an affiliated institution which is paying any applicable article publishing charge ("APC") for Open Access articles. Where the Submitting Author wishes to make the Work available on an Open Access basis (and intends to pay the relevant APC), the terms of reuse of such Open Access shall be governed by a Creative Commons licence – details of these licences and which Creative Commons licence will apply to this Work are set out in our licence referred to above.

Other than as permitted in any relevant BMJ Author's Self Archiving Policies, I confirm this Work has not been accepted for publication elsewhere, is not being considered for publication elsewhere and does not duplicate material already published. I confirm all authors consent to publication of this Work and authorise the granting of this licence.

For peer review only

Governance for injury care systems in Ghana, South Africa and Rwanda: development and pilot testing of an assessment tool

The Equi-Trauma Collaborative

Maria Lisa Odland,[1-3] Abdul-Malik Abdul-Latif,[1,4] Agnieszka Ignatowicz,[1] Abebe Bekele,[5-6] Kathryn Chu,[7-8] Anthony Howard,[9-10] Stephen Tabiri,[11-12] Jean Claude Byiringiro,[13-14] Justine I Davies[1,15,16]

1. Institute of Applied Health Research, University of Birmingham, Birmingham, U, K.
2. Department of Obstetrics and Gynecology, St. Olavs University Hospital, Trondheim, Norway
3. Malawi-Liverpool-Wellcome Trust Research Institute, Blantyre, Malawi
4. Volta Regional Health Directorate, Ghana Health Service, Ho, Ghana
5. University of Global Health Equity, Kigali, Rwanda
6. Department of Surgery, Addis Ababa University, Addis Ababa, Ethiopia
7. Centre for Global Surgery, Department of Global Health, Stellenbosch University, Cape Town, South Africa
8. Department of Surgery, University of Botswana, Gaborone, Botswana
9. Leeds Institute of Rheumatic and Musculoskeletal Medicine, School of Medicine, University of Leeds, Leeds, UK
10. Nuffield Department of Orthopaedics, Rheumatology and Musculoskeletal Sciences, National Institute of Health Research (NIHR) Biomedical Centre, University of Oxford, Headington, Oxford, UK
11. Ghana HUB of NIHR Global Surgery, Tamale, Ghana. Department of Public Health, Tamale Teaching Hospital, Tamale, Ghana
12. Department of Surgery, Tamale Teaching Hospital, Tamale, Ghana
13. University of Rwanda, Kigali, Rwanda
14. Department of Surgery, University Teaching Hospital of Kigali, Kigali, Rwanda
15. Centre for Global Surgery, Department of Global Health, Stellenbosch University, Cape Town, South Africa
16. Medical Research Council/Wits University Rural Public Health and Health Transitions Research Unit, Faculty of Health Sciences, School of Public Health, University of the Witwatersrand, Johannesburg, South Africa

Corresponding author:

Agnieszka Ignatowicz, a.m.ignatowicz@bham.ac.uk, University of Birmingham, Birmingham Clinical Trials Unit College of Medical and Dental Sciences, Birmingham B15 2TT, United Kingdom,+44 (0) 121 414 3344

Keywords: Injuries, Governance, Trauma, Health Policy, Public Health

Word count: 3926 excluding title page, abstrac references, figures and tables.

For peer review only - http://bmjopen.bmj.com/site/about/guidelines.xhtml

Abstract

Objectives: This study aims to evaluate health systems governance for injury care in three sub-Saharan countries from policymakers' and injury care providers' perspectives.

Setting: Ghana, Rwanda and South Africa.

Design: Based on Siddiqi at al.'s framework for governance, we developed an online assessment tool for health system governance for injury with 37 questions covering health policy and implementation under ten overarching principles of Strategic vision, Participation and Consensus orientation, Rule of Law, Transparency, Responsiveness of Institutions, Equity, Effectiveness or Efficiency, Accountability, Ethics, and Intelligence/information. A literature review was also done to support the scoring. We derived scores using two methods - investigator scores and respondent scores.

Participants: The tool was sent out to purposively selected stakeholders, including policymakers and injury care providers in Ghana, Rwanda and South Africa. Data were collected between October 2020 and February 2021.

Primary and secondary outcomes: Investigator-weighted and respondent percentage scores for health system governance for injury care. This was calculated for each country in total and per principle.

Results: Rwanda had the highest overall investigator-weighted percentage score (70%), followed by South Africa (59%). Ghana had the lowest overall investigator score (48%). The overall results were similar for the respondent scores. Some areas, such as Participation and Consensus, scored high in all three countries, whilst other areas, such as Transparency, scored very low.

For peer review only - http://bmjopen.bmj.com/site/about/guidelines.xhtml

Conclusion: In this multi-country governance survey, we provide insight into and evaluation of health system governance for trauma in three low and middle-income countries in Sub-Saharan Africa. It highlights areas of improvement that need to be prioritised, such as transparency, to meet the high burden of trauma and injuries in low and middle-income countries.

For peer review only

For peer review only - http://bmjopen.bmj.com/site/about/guidelines.xhtml

Strengths and limitations

- This is the first study to use an adapted tool to assess health systems governance for injury care in low and middle-income countries.
- We obtained responses from a range of professionals working with trauma care in three different countries in Sub-Saharan Africa.
- A major limitation is that we only had five participants in two of the countries (Rwanda and South Africa) and the low number of respondents could have introduced selection bias.
- If there was no available evidence, the investigators had to weigh the replies from the respondents according to their background, which involved making assumptions about the respondents´ knowledge of the subject.
- This survey tool provides useful insight in the governance of trauma systems in three LMIC countries with different development status.

For peer review only - http://bmjopen.bmj.com/site/about/guidelines.xhtml

Introduction

Injury is a leading cause of disability globally and responsible for more than 5 million deaths each year.[1] Mortality from injury account for more deaths than tuberculosis (TB), malaria and HIV combined[1], and 90% of these deaths occur in low- and middle-income countries (LMICs).[2] Whilst deaths and disability life years (DALYs) lost from many other conditions are in decline, DALYs from injuies remain stubbornly high. Indeed, deaths from injury are predicted to become the leading cause of death by 2030.[3] Despite improvements in road traffic safety in most high-income countries, many LMICs are now having an increasing number of motorised vehicles and road traffic accidents in addition to other common accidents causing injuries such as falls and burns. Still, only a few LMICs have well-defined trauma systems or trauma registries.[4]

The United Nations Development Program defines governance as the exercise of political, economic, and administrative authority in managing a country's affairs at all levels.[5] Governance has long been a critical factor that influences a country's economic growth, social advancement, and general development. It is recognised as especially important for advancing progress towards attaining the Sustainable Development Goals (SDGs) in LMICs.[6] Over recent years, there has been increasing interest in health systems governance with the recognition that good governance leads to better health outcomes for individuals and populations.[6] In 2014 the Lancet-University of Oslo Commission on Global Governance for Health called for a cross-sectoral global action and platform for health governance. This platform may serve as a policy forum to allow the contribution of diverse stakeholders to frame issues, set agendas, and debate policies that affect health and health equity.[7] The World Health Organisation first introduced the term "stewardship" – a part of governance, in

For peer review only

the year 2000, and called for strategic policy frameworks that would allow the incorporation of effective oversight, regulation, incentives, and accountability in health governance.[8]

Health system governance thus involves setting evidence-based shared strategic visions and objectives, in addition to making policies, legislation and deploying resources to ensure the goals and objectives are achieved.[9] However, despite its importance in supporting the delivery of better services and improved health outcomes, little is done to monitor and evaluate health system governance in LMICs.[10-12] Additionally, literature on health system governance around trauma care in LMICs is scarce.[13] Previous studies on health system governance have primarily focused on general health systems functions and particularly on the role of government in governance and the involvement of communities.[14] Moreover, the sparse disease-specific literature that exists focuses on the governance of programs for immunisation[15], TB control[16], mental health care[17-19], and achieving global HIV goals.[20]

Given the prevalence of injuries and the recognised need to invest in health services to provide trauma care, good governance will be essential to ensure that the care provided is of high quality and accessible to those who need it. As part of a larger project that identified barriers in access to quality care for people who have been injured in LMICs[21], we adapted a tool to assess the health system governance for trauma care in three diverse countries in Sub-Saharan Africa – Ghana, Rwanda and South Africa. Our aim was to try to understand the foundations on which to build improved health systems for trauma and injuries in LMICs.

For peer review only

Methods

Study setting

The study was conducted in three LMIC countries in sub-Sahara Africa: Ghana, Rwanda and South Africa which have vastly different development and health systems. Ghana is a lower middle-income country, an estimated population of 30.4 million people (2019), a life expectancy of 63.8 years, and a Gross National Income (GNI) per capita of $2,220.[22] While health service delivery in the country is largely provided by government, private health institutions also provide significant proportion of health services to the population.[23] The National Ambulance Service provides 24-hour pre-hospital care for accidents and emergencies as part of the care provided by the government.[23] It has been estimated that 7.56% of deaths and 7.24% of disability-adjusted life years (DALYs) in Ghana are due to trauma.[22, 24]

Rwanda, a landlocked East African country of 12.6 million people, has a life expectancy of 68.7 years and GNI per capita of $830.[22] It is classified as a low-income country. Around 9% of all deaths and 10% *of* DALYs are due to trauma.[22] Following the near decimation of its health system by the 1994 genocide, the country has taken steps to strengthen it, giving autonomy to District Health Services to serve urban and rural zones.[25] It introduced the Community-Based Health Insurance (CBHI) system in 1999/2000 to provide health insurance to rural populations.[26] However, the health system is still challenged, and deficiencies exist in the provision of quality trauma care.[27]

For peer review only - http://bmjopen.bmj.com/site/about/guidelines.xhtml

South Africa is an upper-middle-income country, with a population of 68.6 million and a life expectancy of 63.8 years. Injuries are estimated to be responsible for 10% of death and 11% *of* DALYs.[22, 24] South Africa has the third biggest economy in Africa and a GNI per capita of $6,040.[22] Most South Africans (84%) access health services through government clinics, whilst the more affluent people go to private hospitals.[28]

Data collection

Building on the framework and tool developed by Siddiqi et al.[6] for assessing the health system governance in developing countries, we developed an assessment tool for injury/trauma health system governance with 37 questions covering health policy and implementation using the ten overarching principles outlined by Siddiqi et al.: Strategic vision, Participation and Consensus orientation, Rule of Law, Transparency, Responsiveness of Institutions, Equity, Effectiveness or Efficiency, Accountability, Ethics, and Intelligence/information (Appendix 1 and Table 1). Adjustments to the original tool were made to tailor the questions to trauma; these were made based on discussions between the authors of this paper. The resultant tool was piloted for acceptability and comprehensibility before use. Data were collected over a five-month period from October 2020 to February 2021 by participants self-completing an online Word or Google form, based on their preference. Participants were requested to select a single response for each question and included a free text field for notes, and provision of evidence to support their responses was encouraged. All responses were imported and analysed in Microsoft Excel.

An extensive review of grey and published literature documents was also done to support the assessment, particularly the scoring.

For peer review only - http://bmjopen.bmj.com/site/about/guidelines.xhtml

Survey respondent selection

Our aim was to recruit participants from health policy or senior leaders in trauma care provision in each country. Given that we expected potential participants to have sound knowledge of the policy and governance context for injury care in their countries, we aimed for a sample size of 5-8 respondents. We contacted potential participants until at least the minimum number was achieved. A combination of purposive and snowball sampling was used to recruit respondents, with potential participants identified with the support of in-country senior researchers within injury care. Emails were sent to potential participants to request their participation in the study; for each invited participant, two further reminders were sent.

Grey literature search

We also searched for and reviewed program documents, policies, annual reports, and standard operating procedures for each country. Searching was done through the websites of government organisations at the national and sub-national level, websites of international organisations, and the Google search engine. The search terms included the country name and trauma policy, trauma law, strategic plan for trauma, injury, injury work plan, injury policy, injury care, trauma care, injury guidelines, trauma guidelines, and combinations of these. There was no restriction on the year of publication.

Scoring

Scoring was done separately for each country. For each principle there were already a set number of questions outlined by Siddiqi et al. to give a maximum score. Responses were awarded points for each question and treated as binary categorical (0 or 1) or ordinal (0, 1 or 2) (see appendices). We derived scores using two methods - investigator-weighted scores

For peer review only - http://bmjopen.bmj.com/site/about/guidelines.xhtml

and respondent scores. For the respondent scores, the mean score across respondents for each question was computed as the average score from the responses for each question. Given that the response rate for each country differed, the denominator (n) varied based on the number of responses: 11 for Ghana, 5 for Rwanda and 5 for South Africa.

Whilst the investigator-weighted scores considered the following to derive a final score for each question: results from respondents, respondents' professional roles, and the availability of evidence from policy documents and the grey literature searches. These investigator scores were derived after discussions between the authors. Consideration of the respondent's professional roles depended on the question asked; more emphasis was given to responses from policymakers rather than trauma care providers for policy-related questions, and more was given to trauma care providers for questions related to service provision. So, for example, if a trauma care provider gave a score of 0, and the policymaker gave a score of 2 on a question related to policy, such as; "*are there legal documents of injury care*?", the question would receive a final score of 2 as the policymaker was more likely to have up-to-date knowledge. If a policy document was available to answer a question definitively, the literature took precedence over respondents. This process was done through discussions between two authors: AMAL and MLO. When there were disagreements, third and fourth investigators served as arbiters (AI and JD).

Both investigator scores and the average respondent scores for each principle were calculated by dividing the achieved score in each country by the total score possible to achieve and multiplying it by 100. Comparisons across countries are described for each of the 10 Principles and overall.

For peer review only - http://bmjopen.bmj.com/site/about/guidelines.xhtml

Table 1. Applying Siddiqi et al. governance framework to trauma care systems in Ghana, Rwanda and South Africa. Questions for each governance principle are in table 3

S/N	Governance principle	Explanation of principles based upon from Siddiqi et al (2008)	Domain captured for trauma care	Maximum score for principle
**1**	Strategic vision	Through an understanding of the historical, cultural and social complexities of society, leaders have a strong sense of direction for the achievement of long and broad health and human development goals.	There is a detailed long term strategic plans to improve trauma care	12
**2**	Participation and consensus orientation	Everyone or interest groups or institutions acting on behalf of everyone should be given the chance to have a say in relation to decisions about health. This is built on the principle of freedom of association and speech as well as capacities to participate constructively. Good governance should be able to mediate between differing opinions among stakeholders on health, policies and procedures in order to reach a mutual understanding that is beneficial for all.	There is stakeholder participation and level of engagement in policy formulation and implementation for trauma	3
**3**	Rule of law	Legal frameworks or policies relating to human rights on health especially should be applied impartially	There is availability and enforcement of laws, guidelines, policies to support trauma care	6
**4**	Transparency	There should be free flow of information on all health matters. There should be enough information available to all to not only monitor but also understand health matters. Processes, institutions and information should be directly accessible to those concerned with them.	There is transparency on commitments to trauma and available information on indicators and other trauma related information for providers (district) involved in local trauma service provision	3

For peer review only

**5**	Responsiveness	Institutions and processes should promptly serve all stakeholders and ensure that their health and non-health needs are met without delays	Trauma systems are responsive to trauma care needs of the population	10
**6**	Equity and inclusiveness	Everyone should have the opportunity to improve or maintain their health and well being	There is equity in access to quality trauma care	8
**7**	Effectiveness and efficiency	Institutions and processes should maximize available resources to render best health care services according to population needs, as well as influence improved health outcomes	There is the existence of organisational capacity including human resource, communication processes to support quality trauma provision	6
**8**	Accountability	People put in positions of trust from government, the private sector and civil society organisations should be accountable to the public and institutional stakeholders. Accountability in this sense varies depending on the type of institution or organisation and whether or not decisions are for internal or external purposes	There is evidence of accountability between service providers and users in the provision of trauma care	3
**9**	Intelligence and information	Essentials for understanding of the health system to guide the implementation of good policies that are based on empirical data to influence the behaviour of different interest groups that support the strategic vison for health.	There is availability of tools and capacity to capture trauma care data	2
**10**	Ethics	Widely accepted principles of health care ethics; non-maleficence, beneficence and justice. This also includes ethics in health care research essential to safeguard the interest and rights of the patients.	There is enforcement of high ethical standards in trauma care provision and research	3
	**Maximum score**			**56**

237

238

Patient and public involvement

It was not appropriate or possible to involve patients or the public in the design, or conduct, or reporting, or dissemination plans of our research.

Results

Table 2 shows a breakdown of the number of respondents from each country and their employment role at the time of completing the survey. Respondents were made up of key officials employed directly by or advising Department or Ministry of Health, trauma care providers (some of whom were also involved in research), and government officials. Thirteen potential respondents were contacted from each country.

Table 2. Breakdown of respondents from each country and their characteristics

S/N	Country	No of potential participants contacted	No of respondents	No of policy respondents	No of trauma care providers
1	Ghana	13	11	3	8
2	Rwanda	13	5	3	2
3	South Africa	13	5	3	2

Appendix table 2 shows the investigator score for each country according to each question and percentage score for each principle and overall for each country. Some of the respondents provided evidence to support their answers such as policy documents and peer-reviewed papers.

Rwanda had the highest overall investigator percentage score (70%) followed by South Africa (59%). Ghana had the lowest overall investigator percentage score (48%) (Table 3). The overall

For peer review only - http://bmjopen.bmj.com/site/about/guidelines.xhtml

257 results were similar for the respondent average percentage score, with Rwanda scoring 39.85
258 (71%) in total, South Africa 31.07 (56%) and Ghana 18.5 (33%) (Appendix Table 3 – with both
259 percentage scores shown for comparison).

260

261 **Table 3 Investigator score for each question and percentage score for each principle and**
262 **overall for Rwanda, Ghana and South Africa, respectively**

Principle	One question out of many questions asked in this principles	Maximum score for questions	Rwanda score	Ghana score	South Africa score
**Strategic vision**	Is there specific mention of trauma in the national health plan or policy? Or are there specific national health policies around trauma care?	12	8 (67%)	4 (33%)	6 (50%)
**Participation and consensus**	What is the level of stakeholder engagement/community inparticipation at the national and provincial level in trauma policy and related interventions?	3	3 (100%)	3 (100%)	3 (100%)
**Rule of law**	Are there guidelines for accreditation of trauma care providers (doctors, nurses, etc) and are these enforced?	6	4 (67%)	5 (83%)	5 (83%)
**Transparency**	Are managers (District Directors of Health, Medical Superintendents of hospitals) evaluated on their health facility or facilities reaching specific targets for trauma care? And if so, are the results of these evaluations available and accessible?	3	3 (100%)	0 (0%)	0 (0%)

For peer review only - http://bmjopen.bmj.com/site/about/guidelines.xhtml

**Responsiveness of institutions**	Is there mandatory reporting of health facility trauma data and is this used to define the burden of injury at a national level?	10	8 (80%)	5 (50%)	5 (50%)
**Equity**	Are there national level financial schemes to ensure the poor who are injured do not have to pay out of pocket direct medical costs of trauma care?	8	3 (38%)	2 (25%)	3 (38%)
**Effectiveness and efficacy**	Is there a national trauma registry (information management for trauma care)? Is it used? In both private and public?	6	5 (83%)	4 (67%)	4 (67%)
**Accountability**	Are there mechanisms to report failing trauma services to policy makers or regulatory authorities?	3	1 (33.3%)	1 (33.3%)	3 (100%)
**Intelligence/information**	Do staff providing trauma services understand what data needs to be captured and do they have the right data capturing tools to enable them to do this?	3	1 (33%)	1 (33%)	1 (33%)
**Ethics**	Are there any standard operating procedures in place to ensure quality and ethical trauma care for injured people?	3	3 (100%)	2 (67%)	3 (100%)
**Overall Total (% maximum overall score)**		**56**	**39 (70%)**	**27 (48%)**	**33 (59%)**

263

264 Considering the investigator scores, Rwanda had the highest scores for each Principle except
265 for Equity. Participation and consensus, in particular had a very high score in Rwanda (100%),
266 whilst the other scores were between 70-80%, apart from Strategic vision (66.7%) and Equity
267 (37.5%). Like Rwanda, South Africa also had high investigator-weighted scores overall but had

For peer review only - http://bmjopen.bmj.com/site/about/guidelines.xhtml

low scores for Strategic vision (50.0%), Equity (37.5%), and Intelligence and Information (50.0%). For Transparency, South Africa had a score of 0%. On the other hand, South Africa had high scores for Participation and consensus orientation (100%), Rule of Law (83.3%), Accountability (100%) and Ethics (100%). Responsiveness of institutions (50.0%) and Effectiveness and efficiency (66.7% and 66.7%) received medium-high scores. Ghana's highest scores were for the Principles of Rule of Law (83.30%), Effectiveness and Efficiency (66.70%) and Ethics (66.70%). However, the scores were low for the other Principles, especially Strategic Vision (33.30%), Transparency (0%), Equity (25%) and Accountability (33.30%). This gave Ghana the overall lowest score in the Governance Assessment for Trauma with an investigator score of 48.20% (see Table 3). The only Principle that received a 100% investigator score in all the countries was Participation and consensus orientation.

Discrepancies between investigator scores and average respondent scores were mostly seen in Ghana, where the overall scores were 33.0% versus 48.20%, respectively (Table 4 and Appendix table). There were fairly large discrepancies for almost all the Principles except for Equity (17.5% versus 25.%), Effectiveness and efficiency (54.7% versus 66.70%), and Accountability (28.70% versus 33.30%) (Appendix 2). The average respondent and investigator percentage scores for each Principle were more similar for the other two countries. In Rwanda, the overall average respondent percentage score was 71.2%, and the average percentage investigator score was 69.6%. Most of the individual Principles had similar respondent percentage scores except for Transparency (60.0% versus 100.0%), Accountability (70.0% versus 33.3%) and Intelligence and Information (80.0% versus 50.0%). In South Africa, the overall average respondent percentage score was 55.5%, and the overall investigator percentage score was 58.9%. Similar to Rwanda, the individual Principle scores were more or

For peer review only - http://bmjopen.bmj.com/site/about/guidelines.xhtml

291 less similar except for those of Strategic vision (38.9% versus 50.0%), Accountability (89.0%
292 versus 100.0%), Intelligence/information (37.5% and 50.0%) and Ethics (89.0% versus 100%).

293

For peer review only

For peer review only - http://bmjopen.bmj.com/site/about/guidelines.xhtml

Table 4 Summary results by principle for each country individually including achieved percentage score (average score and investigator score)

		Rwanda		Ghana		South Africa	
**Principle**	**Maximum scores**	**% achieved (Respondent scores)**	**% achieved (Investigator scores)**	**% achieved (Respondent scores)**	**% achieved (Investigator scores)**	**% achieved (Respondent scores)**	**% achieved (Investigator scores)**
Strategic vision	12	67.1%	66.7%	17.30%	33.30%	38.9%	50.0%
Participation and consensus orientation	3	100.0%	100.0%	46.70%	100%	100.0%	100.0%
Rule of law	6	79.2%	66.7%	63.20%	83.30%	80.5%	83.3%
Transparency	3	60.0%	100.0%	16.30%	0%	0.0%	0.0%
Responsiveness of institutions	10	76.5%	80.0%	35.40%	50%	52.5%	50.0%
Equity	8	36.9%	37.5%	17.50%	25%	40.4%	37.5%
Effectiveness and efficiency	6	89.2%	83.3%	54.70%	66.70%	66.7%	66.7%
Accountability	3	70.0%	33.3%	28.70%	33.30%	89.0%	100.0%
Intelligence/information	2	80.0%	50.0%	36.50%	50%	37.5%	50.0%
Ethics	3	86.7%	100.0%	52.30%	66.70%	89.0%	100.0%
**Overall score**	**56**	**71.2%**	**69.6%**	**33.00%**	**48.20%**	**55.5%**	**58.9%**

For peer review only - http://bmjopen.bmj.com/site/about/guidelines.xhtml

Discussion

To the best of our knowledge, this is the first study that has assessed governance for trauma health systems across multiple countries. The application of our adapted tool revealed strengths and weaknesses in policies and governance of trauma care in Ghana, South Africa and Rwanda. Rwanda achieved fairly high scores (70%), compared to South Africa (59%), and Ghana, which had the lowest score (40%). However, considering the massive burden of injuries and trauma in these countries, our results suggest that there is room for improvement even in the higher-performing countries. At the same time, the gap between the burden of disease and available governance systems and structures was especially seen in Ghana. –The benefits in policies can be ssen when considering the free maternal health care policy which has been vital in ensuring access to health care for women and children, but policies do not exist for injuries and trauma care.[29]

Rwanda scored relatively highly in our survey. This could be because having successfully achieved the MDGs, Rwanda has committed to reducing morbidity and mortality due to injuries.[8, 30] This includes developing policies, training healthcare providers, investing in data collection, and hosting its first national symposium on trauma and injuries in 2019.[31] Hence there has been a focus on improving health systems to care for patients with injuries in the last few years. There is still high mortality and morbidity from injuries in the country. Still, interventions following recent policies and prioritisation of trauma care coupled with efforts to prevent injuries, for example, the recent introduction of speed cameras in urban areas, will likely improve the situation in the coming years.

Given the level of development – being the only upper-middle income country in our study, it is surprising that South Africa had mediocre percentage scores of around 50%. Many LMICs

For peer review only - http://bmjopen.bmj.com/site/about/guidelines.xhtml

have a high burden of injuries and trauma, but South Africa has a relatively large burden of homicide, violence and stabbings.[32] In addition to this, there are other common injuries, such as road traffic accidents and burns. Even though there are programs, services, and ongoing research on this topic, government stewardship and leadership has been absent.[32] Prevention of violence and injury should be a strategic priority for government programmes and policies, and this requires governance and leadership; there are valuable lessons that South Africa can learn from its own excellent governance structures for HIV care.[33]

Overall our results emphasise that more efforts are needed to strengthen overall governance for injury care, considering how crucial governance is to achieve Universal Health Coverage (UCH).[34] Finance cannot be neglected in this process. However, it is also critical to focus on the Principles that were particularly weak in this study (transparency, accountability and intelligence/information), to improve the effectiveness of the health sector.[34] In particular,accountability i and the correction of trauma care underperformance wil remain issues without adequate data generation. Whereas, WHO has developed a trauma registry for LMIC settings that can be tailored to individual country needs, uptake at national levels is lacking, and the use of data collected for health service quality improvement is underdeveloped. Rwanda is the only country in our study that uses the WHO-based trauma registry, and this is only used in five hospitals and without an active quality improvement program, although there are plans to develop this.[30]

Another thing that was evident in our findings was the difference between the investigator and respondent scores. Rwanda had the highest score regardless of the scoring system used, and the overall investigator and respondent scores were similar. However, in the other two countries, the respondent score was lower than the investigator score, especially in Ghana,

For peer review only - http://bmjopen.bmj.com/site/about/guidelines.xhtml

which had the lowest scores altogether. The difference between the respondent and investigator scores suggests that many respondents are unaware of relevant policies/governance structures for trauma in their respective countries. Awareness of these is the first step to using them in order to improve injury and trauma care in the respective countries.[35] Policies are useless if the people in charge of implementing them are unaware of them. According to our survey, this is mostly an issue in Ghana, but also somewhat in South Africa.

This study also revealed some interesting findings in relation to "Participation and consensus orientation", as it was the only principle where all three countries scored 100%. More involvement of stakeholders may improve service delivery and reduce barriers to accessing quality care for injuries after trauma. But, this isn't necessarily the case, as seen in Nigeria, where an increased involvement of stakeholders in the formulation and implementation of TB policies did not necessarily result in good TB control in the community and the health services in the country.[16]

It is likely that multiple components of governance need to be in place – in combination with the awareness of these – for the improvement of healthcare systems. For example, in Ethiopia, improved health system governance was expected to impact critically on scaling up mental health care within primary care facilities.[17] The presence of high-level government support was thought to be a strength along with a National Mental Health Strategy. But unfortunately, there was still a very low baseline awareness of mental health care planning and a lack of leadership and coordination of mental health planning at the national and district level. Indeed, a qualitative study using Siddiqi's framework for mental health governance in South Africa found that facilitating factors to implementing integrated mental health care

For peer review only - http://bmjopen.bmj.com/site/about/guidelines.xhtml

365 were using task-sharing models and establishment of district mental health teams to facilitate
366 the development, and implementation of mental health care plans. The challenges were weak
367 managerial and planning capacity to develop health care at the provincial and district level.
368 All of which speak to the need for knowledge and implementation of governance structures
369 for the improvement of healthcare. Hence to strengthen health care delivery there is a critical
370 need to strengthen leadership and coordination, and implementation at all levels; national,
371 regional, district, and down to individual healthcare facilities. There are valuable lessons from
372 these other disease areas that can be used for governance structures to improve trauma care
373 systems.

374 In this survey, we managed to obtain responses from a range of professionals working with
375 trauma care in three different countries in Sub-Saharan Africa. However, a major limitation is
376 that we only had five participants in two of the countries (Rwanda and South Africa) and found
377 soliciting the involvement of respondents difficult, despite having researchers with links to
378 policy makers leading the study in each country. The low number of respondents could have
379 introduced selection bias. We tried to overcome this bias using an investigator score.
380 However, we may have found different results if we had achieved greater numbers of
381 participants from each country. Nevertheless, our results have face validity, considering that
382 injury care has been an area of focus in Rwanda [31], and Rwanda scored highest in our
383 governance survey. The investigator scores also had their limitations. If there was no available
384 evidence the investigators had to weigh the replies from the respondents according to their
385 background, which involved making assumptions about the respondents´ knowledge of the
386 subject. We did our best to make sure the investigator scores were correct by checking the
387 grey literature and available information. Our scoring system has not been validated and we

peer review only

cannot be certain that the scores were always reflective of the true trauma systems governance of that country, or that one country is doing better than the other. Another limitation was that there was only one question focusing on injury prevention in our survey.

Nevertheless, our study is novel in looking at governance assessment for injuries in LMICs. This survey tool provides useful insight in the governance of trauma systems in three LMIC countries with different development status and provides evidence that governance systems for trauma need to be improved in certain areas in order to face the high burden of injuries in LMICs in the years to come.

Conclusions

In this multi-country governance survey, we have shown that the governance structures for trauma is limited in three different countries in Sub-Saharan Africa; Ghana, Rwanda and South Africa. Some areas, such Participation and consensus, scored high in all three countries whilst other areas such Transparency scored very low. This study provides insight into the governance of trauma systems in these three countries and highlights areas that need to be prioritised in the years to come in order to meet the high burden of trauma and injuries. Assessment of the health systems governance for trauma, as we did in this study, provides evidence that should not only stimulate more research in this area but also support advocacy efforts to advance trauma care systems.

For peer review only - http://bmjopen.bmj.com/site/about/guidelines.xhtml

Contributorship statement

The study was designed by MLO, AA, AI and JD. MLO, AA, KC, AB, ST and JCB contributed to the data collection. MLO, AA, AI and JD did the analysis and interpretation of results. MLO, AA, AI and JD drafted the manuscript. All authors reviewed the results and approved the final version of the manuscript.

Competing interest

The authors declare no conflict of interest.

Funding

This research was funded by the National Institute for Health and Care Research grant number 130036.

Data sharing statement

The data that support the findings of this study are available on reasonable request from the corresponding author, AI.

Ethics statement

The overall study was approved by University of Birmingham Research Ethics Committee, UK (ERN_20- 00880).

Acknowledgements

We would like to acknowledge to all of the participants in our study, who generously shared their time, experiences, and insights with us.

For peer review only

References

1. World Health Organisation. *Injuries and Violence the Facts*. 2014; Available from: https://apps.who.int/iris/rest/bitstreams/668802/retrieve.
2. Gosselin, R.A., et al., *Injuries: the neglected burden in developing countries.* Bull World Health Organ, 2009. **87**(4): p. 246-246a.
3. Mathers, C.D. and D. Loncar, *Projections of global mortality and burden of disease from 2002 to 2030.* PLoS Med, 2006. **3**(11): p. e442.
4. Whitaker, J., et al., *Assessing trauma care systems in low-income and middle-income countries: a systematic review and evidence synthesis mapping the Three Delays framework to injury health system assessments.* BMJ Glob Health, 2021. **6**(5).
5. United Nations. *Human Development Report*. 1997; Available from: http://hdr.undp.org/sites/default/files/reports/258/hdr_1997_en_complete_nostats.pdf.
6. Siddiqi, S., et al., *Framework for assessing governance of the health system in developing countries: gateway to good governance.* Health Policy, 2009. **90**(1): p. 13-25.
7. Ottersen, O.P., et al., *The political origins of health inequity: prospects for change.* Lancet, 2014. **383**(9917): p. 630-67.
8. World Health Organisation. *Everybody's business: Strengthening health systems to improve health outcomes*. 2007; Available from: https://www.who.int/healthsystems/strategy/everybodys_business.pdf.
9. Lewis M. and Petterson G. *Governance in Health Care Delivery*. 2009; Available from: https://poseidon01.ssrn.com/delivery.php?ID=538097071083084007079107106066114005010087014021019030101052032018059029044024014036025095087006090007037092008070123064093068026009089080094025102105080027103111123030016094008003098115064&EXT=pdf&INDEX=TRUEw.
10. Mugisha, J., J. Ssebunnya, and F.N. Kigozi, *Towards understanding governance issues in integration of mental health into primary health care in Uganda.* Int J Ment Health Syst, 2016. **10**: p. 25.
11. Bossert, T.J., *Health systems.* Health Policy Plan, 2012. **27**(1): p. 8-10.
12. Cole, E., *The national major trauma system within the United Kingdom: inclusive regionalized networks of care.* Emergency and Critical Care Medicine, 2022. **2**(2): p. 76-79.
13. Reynolds, T.A., et al., *The Impact of Trauma Care Systems in Low- and Middle-Income Countries.* Annu Rev Public Health, 2017. **38**: p. 507-532.
14. Rosato, M., et al., *Community participation: lessons for maternal, newborn, and child health.* Lancet, 2008. **372**(9642): p. 962-71.
15. Grundy, J., *Country-level governance of global health initiatives: an evaluation of immunization coordination mechanisms in five countries of Asia.* Health Policy Plan, 2010. **25**(3): p. 186-96.
16. Ogbuabor, D.C. and O.E. Onwujekwe, *Governance of tuberculosis control programme in Nigeria.* Infect Dis Poverty, 2019. **8**(1): p. 45.
17. Hanlon, C., et al., *Health system governance to support scale up of mental health care in Ethiopia: a qualitative study.* Int J Ment Health Syst, 2017. **11**: p. 38.

For peer review only - http://bmjopen.bmj.com/site/about/guidelines.xhtml

18. Petersen, I., et al., *Strengthening mental health system governance in six low- and middle-income countries in Africa and South Asia: challenges, needs and potential strategies.* Health Policy Plan, 2017. **32**(5): p. 699-709.
19. Marais, D.L. and I. Petersen, *Health system governance to support integrated mental health care in South Africa: challenges and opportunities.* Int J Ment Health Syst, 2015. **9**: p. 14.
20. Khalid, H. and A.M. Fox, *Political and Governance Challenges to Achieving Global HIV Goals with Injecting Drug Users: The Case of Pakistan.* Int J Health Policy Manag, 2019. **8**(5): p. 261-271.
21. Odland, M.L., et al., *Equitable access to quality trauma systems in low-income and middle-income countries: assessing gaps and developing priorities in Ghana, Rwanda and South Africa.* BMJ Global Health, 2022. **7**(4): p. e008256.
22. The World Bank. *World Bank Open Data*. 2021; Available from: https://data.worldbank.org/.
23. Ghana Health Service Ministry of Health. *Ghana Health Service 2014 Annual Report*. 2014; Available from: https://www.ghanahealthservice.org/downloads/Ghana_Health_Service_2014_Annual_Report.pdf.
24. Institute for Health Metrics and Evaluation. *Country profiles*. Available from: http://www.healthdata.org/results/country-profiles.
25. The DHS Program. *Rwanda: Standard DHS, 2019-20.* 2020.
26. Nyandekwe, M., M. Nzayirambaho, and J.B. Kakoma, *Universal health insurance in Rwanda: major challenges and solutions for financial sustainability case study of Rwanda community-based health insurance part I.* Pan Afr Med J, 2020. **37**: p. 55.
27. Ntakiyiruta, G., et al., *Trauma care and referral patterns in Rwanda: implications for trauma system development.* Can J Surg, 2016. **59**(1): p. 35-41.
28. Mahlahti P. and Dlamini J. *MINIMUM DATA SETS FOR HUMAN RESOURCES FOR HEALTH AND THE SURGICAL WORKFORCE IN SOUTH AFRICA'S HEALTH SYSTEM*. 2015; Available from: https://www.who.int/workforcealliance/031616south_africa_case_studiesweb.pdf.
29. Adu, J., S. Mulay, and M.F. Owusu, *Reducing maternal and child mortality in rural Ghana.* Pan Afr Med J, 2021. **39**: p. 263.
30. Jayaraman, S., et al., *Building Trauma and EMS Systems Capacity in Rwanda: Lessons and Recommendations.* Ann Glob Health, 2021. **87**(1): p. 104.
31. Rosenberg, A., et al., *First Rwanda National Trauma Symposium 2019: Challenges and priorities.* J Glob Health, 2020. **10**(1): p. 010201.
32. Seedat, M., et al., *Violence and injuries in South Africa: prioritising an agenda for prevention.* Lancet, 2009. **374**(9694): p. 1011-1022.
33. Lodge, T., *The politics of HIV/AIDS in South Africa: government action and public response.* Third World Quarterly, 2015. **36**(8): p. 1570-1591.
34. Fryatt, R., S. Bennett, and A. Soucat, *Health sector governance: should we be investing more?* BMJ Glob Health, 2017. **2**(2): p. e000343.
35. Olafsdottir, A.E., et al., *Health systems performance in sub-Saharan Africa: governance, outcome and equity.* BMC Public Health, 2011. **11**: p. 237.

For peer review only

Appendix table 1. Copy of the governance survey

Principle	Questions	Responses (please circle one response for each question)	Maximum score
**Strategic vision**	Is there a specific mention of trauma in the national health plan or policy? Or are there specific national health policies around trauma	Don't know<br>No (0)<br>Yes (1)	**1**
	Is there a national trauma strategy?	Don't know<br>No (0)<br>Yes (1)	1
	What percentage of the country's health budget goes to trauma care?	Don't know<br><10% ((0)<br>10-20% (1)<br>>20% (2)	2
	Is public sector trauma care provided free of charge	Don't know<br>No-not at all (0)<br>Partly (1)<br>Yes-fully (2)	2
	Is there a national insurance scheme that covers trauma care	Don't know<br>No-not at all (0)<br>Partly (1)<br>Yes-fully (2)	2
	Is there a department within the National Ministry of Health dedicated for trauma?	Don't know<br>No department for trauma at all (0)<br>Department for trauma included in another department (1)<br>Stand-alone department for trauma (2)	2
	If the National health plan mentions trauma, or if there are national health policies for trauma care, are there specific objectives	Don't know<br>No (0)	1

For peer review only

	relating to trauma and timelines to achieve them?	Yes (1)	
	If you have answered yes to question 7, have any of those specific trauma objectives been implemented?	Don't know<br>No (0)<br>Yes (1)	1
Scores			12
**Participation and consensus orientation**	Are stakeholders (e.g: NGOs, private companies) involved in health policy formulation for trauma?	Don't know<br>No organisation is involved (0)<br>1-3 organisations are involved (1)<br>> 3 organisations are involved (2)	2
	What is the level of stakeholder engagement/community participation at the national and provincial level in trauma policy and related interventions?	Don't know<br>No involvement at all (0)<br>These groups are involved (1)	1
Scores			3
**Rule of law**	Are there guidelines for accreditation of trauma care providers (doctors, nurses, etc) and are these enforced?	Don't know<br>No guidelines present (0)<br>Guidelines present are not enforced (1)<br>Guidelines present and enforced (2)	2
	Are there laws to enforce a duty of care by hospitals to treat uninsured trauma patients?	Don't know<br>No laws (0)<br>Laws present are not enforced (1)<br>Laws present and enforced (2)	2
	Are there laws to protect against trauma? Example Seat belt laws?	Don't know<br>No laws (0)<br>Laws present are not enforced (1)	2

For peer review only

		Laws present and enforced (2)	
Scores			6
**Transparency**	Is information readily available on financial commitments or allocated budget to trauma care in the public sector at either national or provincial level?	Don't know<br><br>Don't know<br><br>No (0)<br><br>Yes (1)	1
	Are managers (District Directors of Health, Medical Superintendents of hospitals) evaluated on their health facility or facilities reaching specific targets for trauma care? And if so, are the results of these evaluations available and accessible?	Don't know<br><br>No trauma related criteria for assessment (0)<br><br>Trauma related criteria for assessment is used, but not available (1)<br><br>Trauma related criteria for assessment is used and available (2)	2
Scores			3
**Responsiveness of institutions**	Is there mandatory reporting of health facility trauma data and is this used to define the burden of injury at a national level?	Don't know<br><br>No (1)<br><br>Some data (1)<br><br>Adequate/complete data (2)	2
	Are these data used to inform national or provincial policy?	Don't know<br><br>No (0)<br><br>Yes (1)	1
	Are data on clinical post-injury outcomes captured routinely by health facilities?	Don't know<br><br>No (0)<br><br>Yes partially captured (1)<br><br>Yes adequately captured (2)	2

For peer review only

	Are data on post -injury care patient satisfaction routinely captured?	Don't know<br>No (0)<br>Yes partially captured (1)<br>Yes adequately captured (2)	2
	Are these trauma data used in planning services?	Don't know<br>No (0)<br>Yes (1)	1
	What is the level of responsiveness of the health system to non-medical needs of injured people (social needs, mental health needs etc) ?	Don't know<br>Health services cater to or assess only medical needs (1)<br>Health services consider other outcomes relevant to patients, for example their opinions on services provided (2)	2
Scores			10
**Equity**	Are there national level financial schemes to ensure the poor who are injured do not have to pay out of pocket direct medical costs of trauma care?	Don't know<br>No scheme (0)<br>Partial scheme (1)<br>All people are covered (2)	2
	Are there health policies in place to address inequality in access to care for trauma?	Don't know<br>No policies in place (0)<br>Policies in place for general health equity (1)<br>Policies in place specifically for trauma (2)	2
	Are there data which show whether access is equitable or not? And do those data show that access is equitable?	Don't know<br>No data available (0)<br>Data available (1)<br>Data available and show that access is equitable (2)	2

For peer review only

	Is the allocation of trauma care staff to districts and hospitals appropriate and based on needs? Do districts in urban centres get more staff than districts in remote areas?	Don't know<br><br>Less than 2:1 (1)<br><br>More than 2:1 (0)	1
	Is there a mechanism to equitably distribute the budget for health or trauma care?	Don't know<br><br>No (0)<br><br>Yes (1)	1
Scores			8
**Effectiveness and efficiency**	Is there a national trauma registry (information management for trauma care)? Is it used? In both private and public?	Don't know<br><br>No (0)<br><br>Yes (1)	1
	Are there national or provincial guidelines for in -service training of staff on trauma care?	Don't know<br><br>No (0)<br><br>Yes (1)	1
	Is there a pre-hospital referral system (e.g: ambulance service)?	Don't know<br><br>No (0)<br><br>Yes (1)	1
	If pre-hospital referral systems are available, what has been the experience of patients regarding pre hospital referrals?	Don't know<br><br>Poor (0)<br><br>Fair (1)<br><br>Good (2)	2
	Are local transport operators (e.g: taxis) involved in transporting people to hospital?	Don't know<br><br>No (0)<br><br>Yes (1)	1
Scores			6
**Accountability**	Are there mechanisms to report failing trauma services to policy makers or regulatory authorities	Don't know<br><br>No (0)<br><br>Yes (1)	1
	Are there any mechanisms of correcting under performance of trauma services?	Don't know<br><br>No mechanisms (0)	2

For peer review only

		Mechanisms of reporting but no onwards mechanisms to improve quality (1)<br><br>Mechanisms of reporting and mechanisms to improve quality (2)	
Scores			3
**Intelligence/Information**	Do staff providing trauma services understand what data needs to be captured and do they have the right data capturing tools to enable them do this?	Don't know<br><br>No understanding and no tools (0)<br><br>Understanding but no or limited tools (1)<br><br>Understanding and useful tools (2)	2
Scores			2
**Ethics**	Is there any policy available for regulating trauma related research?	Don't know<br><br>No (0)<br><br>Yes (1)	1
	Are there any standard operating procedures in place to ensure quality and ethical trauma care for injured people?	Don't know<br><br>No (0)<br><br>Yes (1)	1
	Are there mechanisms in place in institutes for enforcing high ethical standards in the treatment of trauma patients	Don't know<br><br>No (0)<br><br>Yes (1)	1
Scores			3
Overall maximum score			56

Appendix 2 Investigator score for each question and percentage score for each principle and overall for Rwanda, Ghana and South Africa respectively

Principle	Questions	Maximum score for question	Rwanda score	Ghana score	South Africa score
**Strategic vision**	Is there specific mention of trauma in the national health plan or policy? Or are there specific national health policies around trauma care?	1	1	1	1
	Is there a national trauma strategy?	1	1	0	0
	What percentage of the country's public health budget goes to trauma care?	2	0	0	2
	Is public sector trauma care provided free of charge?	2	1	1	1
	Is there a national insurance scheme that covers trauma care?	2	2	1	1
	Is there a department within the National Ministry of Health dedicated for trauma?	2	1	1	1
	If the National health plan mentions trauma, or if there are national health policies for trauma care, are there specific objectives relating to trauma and timelines to achieve them?	1	1	0	0
	If you have answered yes to question 7, have any of those specific trauma objectives been implemented?	1	1	0	0
**Score for Principle (% of total possible)**		**12**	**8 (67%)**	**4 (33%)**	**6 (50%)**
**Participation and consensus**	Are stakeholders (e.g: NGOs, private companies) involved in	2	2	2	2

For peer review only

	What is the level of stakeholder engagement/community participation at the national and	1	1	1	1
**Score for Principle (% of total possible)**		**3**	**3 (100%)**	**3 (100%)**	**3 (100%)**
**Rule of law**	Are there guidelines for accreditation of trauma care	2	2	2	1
	Are there laws to enforce a duty of care by hospitals to treat uninsured	2	0	2	2
	Are there laws to protect against trauma? Example Seat belt laws?	2	2	1	2
**Score for Principle (% of total possible)**		**6**	**4 (67%)**	**5 (83%)**	**5 (83%)**
**Transparency**	Is information readily available on financial commitments or allocated budget to trauma care in the public sector at either national or provincial level?	1	1	0	0
	Are managers (District Directors of Health, Medical Superintendents of hospitals) evaluated on their health facility or facilities reaching specific targets for trauma care? And if so, are the results of these evaluations available and accessible?	2	2	0	0
**Score for Principle (% of total possible)**		**3**	**3 (100%)**	**0 (0%)**	**0 (0%)**
**Responsiveness of institutions**	Is there mandatory reporting of health facility trauma data and is	2	2	1	1
	Are these data used to inform national or provincial policy?	1	1	1	1
	Are data on clinical post-injury outcomes captured routinely by	2	2	1	0
	Are data on post-injury care patient satisfaction routinely captured?	2	0	0	0
	Are these trauma data used in planning services?	1	1	1	1
	What is the level of responsiveness of the health system to non-medical needs of injured people (social needs, mental health needs etc)?	2	2	1	2

For peer review only

**Score for Principle (% of total possible)**		**10**	**8 (80%)**	**5 (50%)**	**5 (50%)**
**Equity**	Are there national level financial schemes to ensure the poor who are injured do not have to pay out	2	2	1	2
	Are there health policies in place to address inequality in access to care for trauma?	2	1	1	1
	Are there data which show whether access is equitable or not? And do those data show that access is equitable?	2	0	0	0
	Is the allocation of trauma care staff to districts and hospitals appropriate and based on needs? Do districts in urban centres get more staff than districts in remote areas?	1	0	0	0
	Is there a mechanism to equitably distribute the budget for health or trauma care?	1	0	0	0
**Score for Principle (% of total possible)**		**8**	**3 (38%)**	**2 (25%)**	**3 (38%)**
**Effectiveness and efficacy**	Is there a national trauma registry (information management for trauma care)? Is it used? In both private and public?	1	1	0	0
	Are there national or provincial guidelines for in-service training of staff on trauma care?	1	1	1	1
	Is there a pre-hospital referral system (e.g: ambulance service)?	1	1	1	1
	If pre-hospital referral systems are available, what has been the experience of patients regarding pre-hospital referrals?	2	1	1	2
	Are local transport operators (e.g: taxis) involved in transporting	1	1	1	0
**Score for Principle (% of total possible)**		**6**	**5 (83%)**	**4 (67%)**	**4 (67%)**

For peer review only

**Accountability**	Are there mechanisms to report failing trauma services to policy	1	0	1	1
	Are there any mechanisms of correcting under performance of	2	1	0	2
**Score for Principle (% of total possible)**		**3**	**1 (33.3%)**	**1 (33.3%)**	**3 (100%)**
**Intelligence/information**	Do staff providing trauma services understand what data needs to be captured and do they have the right [illegible]	3	1	1	1
**Score for Principle (% of total possible)**		**3**	**1 (33%)**	**1 (33%)**	**1 (33%)**
**Ethics**	Is there any policy available for regulating trauma related research?	1	1	1	1
	Are there any standard operating procedures in place to ensure quality and ethical trauma care for injured people?	1	1	0	1
	Are there mechanisms in place in institutes for enforcing high ethical standards in the treatment of trauma patients?	1	1	1	1
**Score for Principle (% of total possible)**		**3**	**3 (100%)**	**2 (67%)**	**3 (100%)**
**Overall Total (% maximum overall score)**		**56**	**39 (70%)**	**27 (48%)**	**33 (59%)**

For peer review only

Appendix table 3 Summary results by principle for each country individually including average score, investigator score, maximum score possible for each question and achieved percentage score (average score and investigator score).

		Rwanda				Ghana				South Africa			
**Principle**	**Maximum scores**	**Respondent scores (n, average)**	**Investigator scores (n)**	**% achieved (Respondent scores)**	**% achieved (Investigator scores)**	**Respondent scores (n, average)**	**Investigator scores (n)**	**% achieved (Respondent scores)**	**% achieved (Investigator scores)**	**Respondent scores (n, average)**	**Investigator scores (n)**	**% achieved (Respondent scores)**	**% achieved (Investigator scores)**
Strategic vision	12	8.05	8	67.1%	66.7%	2.07	4	17.30%	33.30%	4.67	6	38.9%	50.0%
Participation and consensus orientation	3	3	3	100.0%	100.0%	1.4	3	46.70%	100%	3	3	100.0%	100.0%
Rule of law	6	4.75	4	79.2%	66.7%	3.16	5	63.20%	83.30%	4.83	5	80.5%	83.3%
Transparency	3	1.8	3	60.0%	100.0%	0.49	0	16.30%	0%	0	0	0.0%	0.0%
Responsiveness of institutions	10	7.65	8	76.5%	80.0%	3.54	5	35.40%	50%	5.25	5	52.5%	50.0%
Equity	8	2.95	3	36.9%	37.5%	1.4	2	17.50%	25%	3.23	3	40.4%	37.5%
Effectiveness and efficiency	6	5.35	5	89.2%	83.3%	3.28	4	54.70%	66.70%	4	4	66.7%	66.7%
Accountability	3	2.1	1	70.0%	33.3%	0.86	1	28.70%	33.30%	2.67	3	89.0%	100.0%
Intelligence/information	2	1.6	1	80.0%	50.0%	0.73	1	36.50%	50%	0.75	1	37.5%	50.0%
Ethics	3	2.6	3	86.7%	100.0%	1.57	2	52.30%	66.70%	2.67	3	89.0%	100.0%
**Overall score**	**56**	**39.85**	**39**	**71.2%**	**69.6%**	**18.5**	**27**	**33.00%**	**48.20%**	**31.07**	**33**	**55.5%**	**58.9%**

For peer review only

For peer review only
